# Physical activity and cognitive function in middle-aged adults: a cross-sectional analysis of the PATH through life study

**DOI:** 10.3389/fpsyg.2023.1022868

**Published:** 2023-08-24

**Authors:** Clare Quinlan, Ben Rattray, Disa Pryor, Joseph M. Northey, Nicolas Cherbuin

**Affiliations:** ^1^UC Research Institute for Sport and Exercise, University of Canberra, Canberra, ACT, Australia; ^2^Discipline of Sport and Exercise Science, Faculty of Health, University of Canberra, Canberra, ACT, Australia; ^3^Faculty of Health, Charles Darwin University, Darwin, NT, Australia; ^4^Centre for Research on Ageing, Health and Wellbeing, Australian National University, Canberra, ACT, Australia

**Keywords:** physical activity, cognitive function, middle-adulthood, objectively-measured physical activity, self-reported physical activity

## Abstract

**Objectives:**

Investigate the independent associations of objectively measured or self-reported physical activity at different intensities with cognitive performance in middle-aged adults.

**Design:**

Cross-sectional.

**Methods:**

156 middle-aged adults (age: 40.6 ± 1.5, 58.3% female) participated in the physical activity sub-study of the Personality and Total Health through life (PATH) project. Physical activity was measured objectively with the SenseWear™ armband (SWA), worn for seven consecutive days, and measured *via* self-report with a Physical Activity Recall survey (PAR). Cognitive performance was assessed with the Symbol Digit Modalities Test, the Digit Span Backwards, and an Immediate and Delayed Recall task. Associations between physical activity intensity and cognitive function were investigated in general linear models, controlling for age, sex, and education.

**Results:**

Neither objectively measured nor self-reported physical activity were associated with cognitive function at light-, moderate-, vigorous-, or combined moderate-to-vigorous intensity in this cohort of well educated, healthy middle-aged adults. Sensitivity analyses with additional moderators (e.g., body mass index, hypertension, alcohol intake) and the use of composite cognitive measures did not alter the results.

**Conclusion:**

In this cohort of middle-aged adults, objectively measured and self-reported physical activity do not appear to be associated with cognitive function. Longitudinal follow-ups utilising objective physical activity measures may be important in determining the impact of mid-life behaviours on the trajectory of cognitive changes into older age.

## Introduction

Preventative measures targeted at reducing age-related cognitive decline are crucial for preventing diseases such as dementia (Norton et al., 2014; Shatenstein and Barberger-Gateau, 2015) and maintaining quality of life (Pusswald et al., 2015). Physical activity has been shown to reduce the incidence of dementia risk factors (Erickson et al., 2019), and to positively influence cognitive function (Northey et al., 2017; Liu-Ambrose et al., 2018). Longitudinal evidence indicates that cognition begins to decline from early to middle-age (Park et al., 2002; Singh-Manoux et al., 2012). Higher cognitive performance and physical activity during this life stage may offer protection against later life declines (Livingston et al., 2020). However, we have limited understanding of how physical activity relates to cognition during middle-adulthood. It is critical that we better understand which physical activity dose characteristics, such as volume, frequency, intensity, or duration, contribute most to its protective effects, to adequately inform interventions targeting risk reduction. Components of dose of physical activity must be considered when designing interventions for the specific health outcome being targeted. However, the dose required to influence benefit differs by outcome (Wasfy and Baggish, 2016) and currently, the specifics of physical activity dose required to influence middle-aged adults’ cognitive performance are not well understood (Erickson et al., 2019).

Cross-sectional investigations of middle-aged adults have demonstrated that greater physical activity levels are associated with higher scores on cognitive tasks (Singh-Manoux et al., 2005; Winneke et al., 2012; Berchicci et al., 2013; Erickson et al., 2013; Spartano et al., 2019). Encouragingly, higher self-reported levels of total physical activity during middle-adulthood have been associated with reduced cognitive decline in later life (Chang et al., 2010; Iso-Markku et al., 2016). This has been observed across cognitive domains including processing speed, memory, and executive function (Chang et al., 2010). However, these studies have relied predominantly on self-reported physical activity and have focussed on total physical activity or time spent engaging in moderate to vigorous physical activity (MVPA) (Singh-Manoux et al., 2005; Chang et al., 2010; Winneke et al., 2012; Berchicci et al., 2013; Erickson et al., 2013; Iso-Markku et al., 2016). Cross-sectional investigations in middle-aged adults utilising objective measures of physical activity have only investigated combined MVPA, providing mixed results (Vásquez et al., 2017; Spartano et al., 2019). Spartano et al. (2019) observed that achieving 10–21 min of MVPA per day was associated with better executive function, and reported a dose–response association between MVPA and verbal memory (Spartano et al., 2019). However, Vásquez et al. (Vásquez et al., 2017) observed no benefit of higher levels of MVPA on cognitive function. As the potential of light- (Chastin et al., 2019; Spartano et al., 2019) and vigorous-intensity (Brown et al., 2012; Iso-Markku et al., 2015) physical activity to improve health outcomes becomes more evident, and the differential effect of intensity on cognition is questioned (Erickson et al., 2019) the need to break down physical activity behaviours accurately into intensity brackets becomes more pertinent.

Another primary consideration in understanding physical activity dose requirements for cognitive health is that self-reported physical activity may not correlate well with objective measures due to participants’ inability to accurately report the intensity or duration of physical activity, particularly in relation to ancillary activity (Timperio et al., 2003; Prince et al., 2008; Banda et al., 2010; Schuna et al., 2013; Northey et al., 2016; Quinlan et al., 2020). A systematic review demonstrated that self-reported physical activity is generally less reliable than direct measures as intensity increases (Prince et al., 2008). Despite these known issues, self-reported physical activity measures are commonly used due to their ease of use and cost-effectiveness (Ainsworth, 2009). Self-report methods may introduce errors in measuring actual physical activity characteristics, masking the optimal dose of physical activity required to improve or maintain cognitive function in ageing (Spartano et al., 2019). It is also possible that inter-individual variability of other factors such as personality, health status, cultural background, or educational attainment influence how physical activity is reported. The independent associations of self-reported physical activity levels and cognitive outcomes may not reflect the actual level of physical activity required to achieve optimal benefits or that other underlying factors may produce the associations reported in the literature.

Objectively accounting for the intensity of physical activity and comparing how associations with cognitive performance may differ from those measured by self-reported physical activity could inform more specific guidelines for physical activity for healthy ageing. Therefore, the current cross-sectional study aimed to investigate the independent associations of objectively measured physical activity or self-reported physical activity, subset by intensities of light-, moderate-, and vigorous-intensity, as well as MVPA with cognitive function. The cognitive tasks predominantly assessed domains of working memory, processing speed, and executive functions. These cognitive functions are susceptible to age-related declines (Salthouse, 1996; Borella et al., 2008) and enhanced through physical activity (Nouchi et al., 2014; Zach and Shalom, 2016). The secondary aim was to compare how associations between physical activity and cognitive performance might differ between measurement type.

## Methods

Participants were recruited from the Personality and Total Health through life (PATH) project, described in detail elsewhere (Anstey et al., 2012). At its inception (1999–2000), 7,485 participants were invited to participate based on three age groups: the ‘20+’ age group (20–24 years), the ‘40+’ age group (40–44 years), and the ‘60+’ age group (60–64 years). Each cohort has been followed up every 4 years since the initial testing in waves.

**Figure 1:**
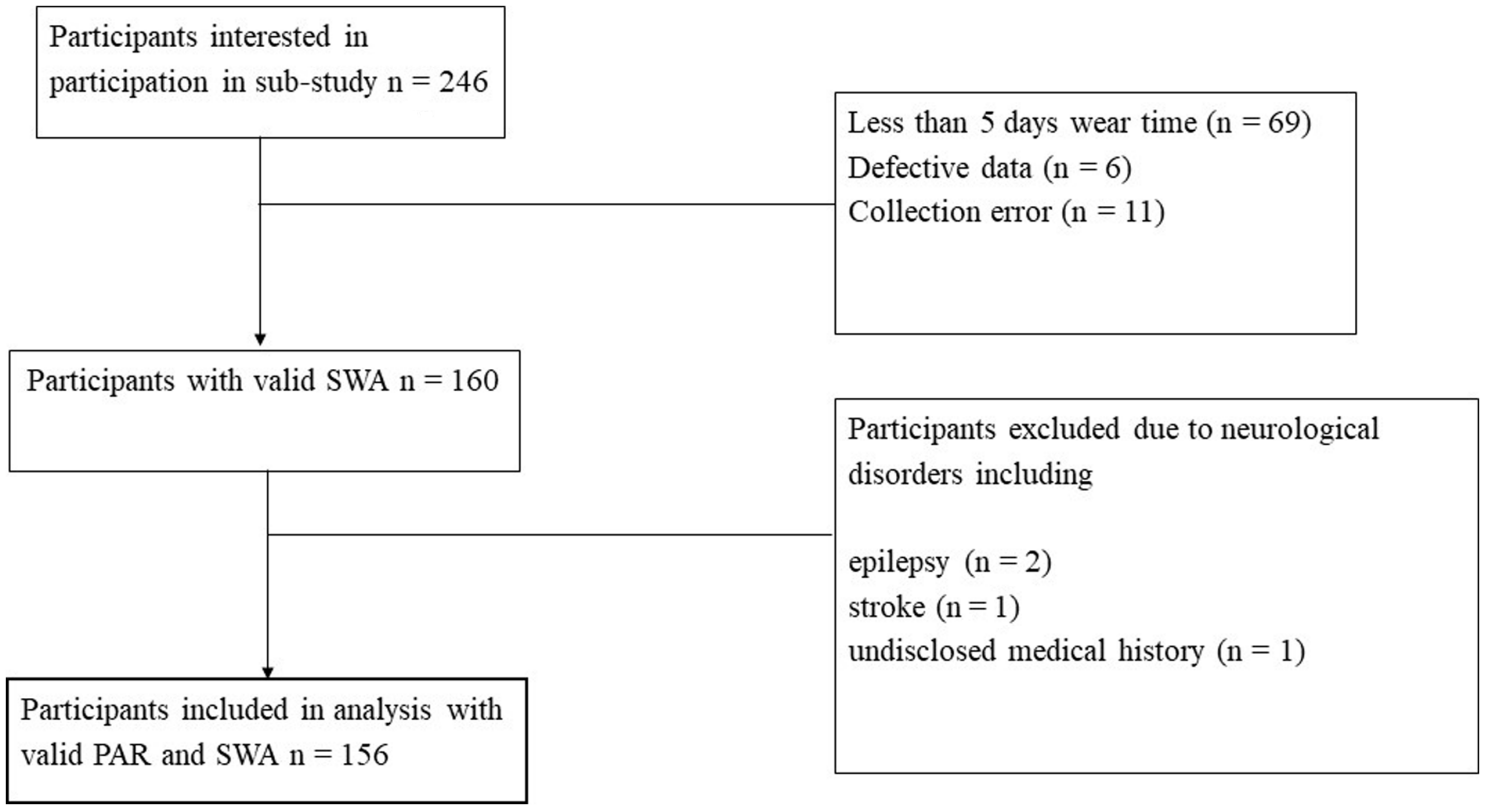
Flow of participants through the study. SWA, SenseWear Armband™; PAR, Physical Activity Recall Survey.

This investigation focuses on the ‘20+’ age group, now aged 37–43, who completed their fifth wave of data collection. Of the 2,404 participants randomly selected at baseline (wave 1), 1,409 returned at wave 5 to complete an online survey, including demographic and health-focused questions, and the completion of the Physical Activity Recall Survey (PAR). Of these participants, 1,259 participated in in a face-to-face interview where a battery of tests were repeated from previous waves, including four cognitive tasks. At the face-to-face interview, 246 participants consented to participate in the current sub-study, run in wave 5 only, and were provided with a SenseWear Armband™ (SWA; BodyMedia, PA, United States) to wear continuously for 7 days. Participants (*n* = 69) were excluded if they had less than five valid days of SWA data (>20 h on the body each day) or did not have valid data for two weekend days (Scheers et al., 2012). Seventeen participants were removed from the analysis due to an error with the SWA. A total of 160 participants had valid PAR data, SWA data, and complete cognitive data. Participants were excluded from analysis if they had reported a previous stroke (*n* = 1), Parkinson’s disease (*n* = 0), or epilepsy (*n* = 2), in line with previous studies using the PATH cohort (Bielak et al., 2014; Northey et al., 2019), or if they were missing components of the PATH survey (*n* = 1), as displayed in Figure 1. The current study was approved by the Australian National University Human Research Ethics Committee, and all participants provided written informed consent (Human Ethics Protocol 2016/445).

Self-reported physical activity was assessed using the PAR. Adapted from the Whitehall II study (Sabia et al., 2014), the PAR requires participants to report time spent (hours:mins/week) engaging in light- (e.g., walking, housework) moderate- (e.g., cycling, swimming) or vigorous-intensity physical activity (e.g., running, sports) over the preceding seven-day period. Subsequently, time spent in moderate to vigorous physical activity (MVPA) was calculated. Total physical activity (MET: min∙week^−1^) was also calculated by combining each physical activity intensity duration by its MET-value (Northey et al., 2019). For the PAR, Total physical activity (MET: min∙week^−1^) was calculated with the formula MET: min∙week^−1^ = (1.5 x light min∙week^−1^) + (3 x moderate min∙week^−1^) + (6 x vigorous min∙week^−1^) (Australian Institue of Health and Welfare, 2003; Northey et al., 2019).

Objectively measured physical activity was assessed using the SWA provided to participants during the face-to-face interview. Providing more accuracy than an accelerometer alone (Welk et al., 2007), the SWA incorporates a tri-axial accelerometer along with heat-flux, skin temperature, near-body ambient temperature, and galvanic skin responses (Liden et al., 2002; Bai et al., 2016). Previous work has shown associations between the SWA and the PAR in the current population (Quinlan et al., 2021). The armband was fitted over the triceps muscle group of the participants left arm. Participants were instructed to wear it for seven consecutive days except during water submersion or showering. The SWA was set to collect data at one-minute intervals. Data were downloaded to the proprietary processing software (SenseWear™ Pro version 8.1, BodyMedia, PA) and combined with participant demographic data on height, weight, smoking status, sex, and handedness, to calculate energy expenditure (Liden et al., 2002). Minute-by-minute data were coded by intensity; classified into MET-values of sedentary (< 1.50 METs), light (1.50–2.99 METs), moderate (3.00–5.99 METs), or vigorous (> 6.00 METs). Weekly totals (mins∙week^−1^) were calculated for light-, moderate-, vigorous-intensity, and MVPA to be used as outcomes in subsequent analysis.

Cognitive function was assessed using four tasks. The immediate and delayed recall of the first list of the California Verbal Learning Test were used to assess episodic memory (Delis et al., 1988). The delayed recall component was conducted after an interference task. Both tasks were scored as the number of correct items recalled. The Symbol Digit Modalities Test (SDMT) involves divided attention, visual scanning, and tracking and was used to assess speed-of-processing (Smith, 2007), and was scored by summing the number of correct responses within 90 s. Working memory was assessed using the Digit Span Backwards Task (DSBT), a subset of the Wechsler Memory scale (Wechsler, 1945). The DSBT was scored as the total number of strings of digits of increasing length participants could correctly recite backwards. The scores of each cognitive task were converted to z-scores. A composite score for fluid intelligence was created by combining the z-scores of all tasks and dividing them by four, and for memory combining the z-scores of the immediate and delayed recall tasks divided by two. The composite scores were used in the sensitivity analysis.

Sociodemographic and health information, including age, sex, height, weight, education level, diabetes status, hypertension, and alcohol consumption, were assessed *via* self-report. From the reported height and weight, Body Mass Index (BMI) (weight (kg)/ height^2^ (m)) was calculated. Education level was assessed as the highest qualification achieved. The included covariates are consistent with those utilised in the analysis of PATH data, and have been associated with cognitive decline (Livingston et al., 2020). From 11 initial categories, four education categories were created for use in subsequent analysis: completion of year 10 or equivalent, completion of year 12 or equivalent, completion of vocational training, and completion of a university degree. Hypertension was determined if a participant reported being told by a doctor that they had high blood pressure and were currently on blood pressure medication. The Alcohol Use Disorder Identification Test was used to assess alcohol intake (Saunders et al., 1993).

Statistical analysis was conducted in R (version 3.6.0, R Core Team (2020), Vienna, Austria), using RStudio (RStudio Team (2019), Boston, MA). Descriptive data are presented for included participants as means and 95% confidence intervals for continuous variables and percentages for categorical variables. Separate univariate general linear models were produced to investigate the relationship between cognitive performance and physical activity. For each model, the cognitive outcome was the dependant variable, and the independent variable was the PAR or SWA based outcome, controlled for the covariates age, sex, and education (Sabia et al., 2014). Age was mean centred at 40.6 years. No interaction terms between the independent variable and covariates age, sex, and education were included in the initial models. In the second model, additional moderator terms were added. Finally, interaction terms between the independent variable and the moderators were added. The presence of non-linear relationships was investigated through the addition of a quadratic term into each model, all of which were non-significant. Sensitivity analyses were conducted using general linear models to investigate the effect of physical activity measured by the SWA, when separated into quartiles, on cognitive function. The sensitivity analysis was conducted controlling for age, sex, and education. Sensitivity analysis was conducted as Spartano et al. (2019) analysed data in four categories, and allows for a more specific comparison to be drawn. For all models, visual inspection of QQ-plots showed acceptable distribution. To reduce the risk of Type I errors associated with multiple comparisons, a Simes-Benjamini-Hochberg false discovery rate adjustment was applied to the value of ps generated from the general linear models (Benjamini and Hochberg, 1995). Statistical significance was set at adjusted *p* < 0.05.

## Results

One hundred and fifty-six participants (58.3% female) aged between 38 and 43 years with a mean age of 40.6 (± 1.5) years were included in the current study (Table 1). Participants who were included in the analyses were slightly older compared to the broader PATH cohort (40.6 ± 1.5 vs. 40.3 ± 1.5 years *p* = 0.03) but had a similar level of education, and included a similar proportion of females (*p*’s > 0.05).

**Table 1:** Physical activity, demographic characteristics and cognitive performance of participants included in analysis.

Demographics				
	All	Female	Male	p^*^
Age, y (SD)	40.6 (1.5)	40.7 (1.5)	40.6 (1.6)	0.70
Range	38–43	38–43	38–43	
BMI, kg^.^m^2^ (SD)	26.6 (5.9)	26.3 (6.8)	26.9 (4.5)	0.50
Female, *n* (%)	91 (58.3)			
Education *n* (%)				
Year 10	8 (5.1)	6 (6.6)	2 (3.1)	0.30
Year 12	18 (11.5)	6 (3.6)	12 (18.5)	0.03
Vocational	37 (23.7)	19 (20.9)	18 (27.7)	0.34
University degree	93 (59.6)	60 (65.9)	33 (50.8)	0.06
Diabetes *n* (%)	2 (1.3)	2 (2.2)	0 (0.0)	0.16
Alcohol consumption *n* (%)				
Abstain	5 (3.2)	4 (4.4)	1 (1.5)	0.28
Occasional	43 (27.6)	28 (30.8)	15 (23)	0.28
Light	71 (45.5)	33 (36.3)	38 (58.5)	0.01
Medium	22 (14.1)	14 (15.4)	8 (12.3)	0.58
Hazardous	13 (8.3)	12 (13.2)	1 (1.5)	0.003
Harmful	2 (1.3)	0 (0.0)	2 (3.1)	0.16
Hypertension *n* (%)	7 (4.5)	3 (3.3)	4 (6.15)	0.63
Physical activity measured by the PAR (mean, 95% CI)				
Light PA (min∙week-1)	420 (351 to 490)	410 (334 to 485)	436 (305 to 566)	0.73
Moderate PA (min∙week-1)	196 (173 to 220)	189 (160 to 218)	207 (166 to 247)	0.48
Vigorous PA (min∙week-1)	159 (129 to 189)	133 (89 to 178)	194 (157 to 232)	0.03
MVPA (min∙week-1)	355 (313 to 397)	322 (379 to 265)	401 (339 to 463)	0.06
TOTAL PA (MET:mins∙week-1)	2,171(1912 to 2,429)	1979 (1,643 to 2,314)	2,439 (2031 to 2,848)	0.08
Physical activity measured by the SWA (mean, 95% CI)				
Light PA (min∙week^−1^)	1727 (1,628 to 1825)	1795 (1,658 to 1933)	1,631 (1,494 to 1767)	0.09
Moderate PA (min∙week^−1^)	717 (634 to 800)	563 (481 to 646)	932 (783 to 1,081)	<0.001
Vigorous PA (min∙week^−1^)	68 (51 to 85)	47 (28 to 66)	96 (65 to 127)	0.01
MVPA (min∙week-1)	785 (695 to 874)	611 (518 to 703)	1,028 (872 to 1,184)	<0.001
TOTAL PA (MET:mins∙week^−1^)	6,742 (6,252 to 7,233)	5,990 (5,422 to 6,558)	7,795 (6,977 to 8,613)	<0.001
Cognitive function (mean, 95% CI)				
Immediate recall (n recalled)	9.3 (8.9 to 9.7)	9.7 (9.2 to 10.2)	8.8 (8.2 to 9.3)	<0.001
Delayed recall (n recalled)	8.4 (7.9 to 8.8)	8.9 (8.3 to 9.5)	7.7 (7.1 to 8.4)	<0.001
SDMT(n correct)	67.0 (65.5 to 68.6)	66.8 (64.9 to 68.6)	67.4 (64.9 to 70.0)	<0.001
DSBT (n recalled)	6.6 (6.3 to 6.9)	6.7 (6.3 to 7.1)	6.6 (6.0 to 7.1)	<0.001

Descriptive data are presented as means and standard deviations for continuous variables, and percentages for categorical variables. ^*^for difference between males and females. PAR, Physical Activity Recall survey; SWA, SenseWear Armband; PA, Physical activity; MVPA, moderate-to-vigorous PA; BMI, Body Mass Index; CI, Confidence Interval; SDMT, Symbol Digit Modalities Task; DSBT, Digit Symbol Backwards Task.

As displayed in Table 2, physical activity was not associated with cognitive function at any intensity outcome for the PAR or SWA when controlled for age, sex, and education (full models displayed in Supplementary Tables S1a,b). Models that included other potential moderating variables, including BMI (mean centred at 26.6 kg^.^m^2^), hypertension, diabetes status, and alcohol consumption, were also evaluated for the PAR and SWA, which did not alter the results (Supplementary Tables S2a,b). In addition, analysis with the composite score of memory and fluid intelligence did not change the results (Supplementary Tables S3a,b). Further analysis with interaction terms did not alter the results for individual cognitive tasks (Supplementary Tables S4a,b) or composite scores (Supplementary Tables S5a,b). As displayed in Supplementary Tables S6–S9, the models used to investigate the effect of physical activity measured by the SWA, when split into quartiles, resulted in non-significant models.

**Table 2:** The association between physical activity, measured by the Physical Activity Recall Survey and the SenseWear Armband™, and cognitive function outcomes.

		Immediate recall		Delayed recall		SDMT		DSBT	
		β ± SE	*p*^#^	β ± SE	*p*^#^	β ± SE	*p*^#^	β ± SE	*p*^#^
Light PA (min∙week^−1^)	SWA	0.02 ± 0.08	0.78	0.07 ± 0.08	0.58	−0.02 ± 0.08	0.88	−0.01 ± 0.08	0.94
	PAR	−0.05 ± 0.08	0.54	−0.04 ± 0.08	0.78	−0.06 ± 0.08	0.74	0.05 ± 0.08	0.63
Moderate PA (min∙week^−1^)	SWA	0.01 ± 0.08	0.89	−0.01 ± 0.08	0.92	0.07 ± 0.09	0.87	0.08 ± 0.09	0.38
	PAR	0.04 ± 0.08	0.59	0.06 ± 0.08	0.67	0.05 ± 0.08	0.80	0.02 ± 0.08	0.86
Vigorous PA (min∙week^−1^)	SWA	0.09 ± 0.08	0.33	0.06 ± 0.08	0.66	0.08 ± 0.09	0.82	0.06 ± 0.09	0.56
	PAR	−0.04 ± 0.08	0.66	−0.03 ± 0.08	0.80	0.10 ± 0.08	0.80	−0.01 ± 0.08	0.89
MVPA (min∙week^−1^)	SWA	0.03 ± 0.08	0.74	0.003 ± 0.08	0.96	0.08 ± 0.09	0.92	0.09 ± 0.09	0.37
	PAR	−0.001 ± 0.08	0.99	0.01 ± 0.08	0.92	0.10 ± 0.08	0.83	0.002 ± 0.08	0.98
TOTAL PA (MET:mins∙week^−1^)	SWA	0.05 ± 0.08	0.51	0.05 ± 0.08	0.81	0.08 ± 0.09	0.88	0.10 ± 0.08	0.38
	PAR	−0.03 ± 0.08	0.68	−002 ± 0.08	0.90	0.06 ± 0.08	0.80	0.02 ± 0.08	0.85

Model: age, sex, education. PAR, Physical Activity Recall survey; SWA, SenseWear Armband™; SDMT, Symbol Digit Modalities Test; DSBT, Digit-Span Backwards Task; PA, Physical activity; MVPA, moderate-to-vigorous physical activity. β: standardised coefficient. ^#^ adjusted value of *p*. Full models displayed in Supplementary Tables S1a,b.

## Discussion

The association between physical activity and cognitive function in middle-aged adults has previously focused on MVPA or total PA, yielding mixed results. This cross-sectional investigation is, to our knowledge, the first to examine the associations between cognitive function and both self-reported and objectively measured physical activity, stratified into multiple intensities in a middle-aged cohort. Informing on the independent associations of MVPA and total PA and light-, moderate-, and vigorous-intensity on cognitive function may provide more insight into the dose–response relationship of PA with cognitive function. However, we did not find evidence of an association between self-reported or objectively measured physical activity of any intensity with any of the cognitive function tasks, predominately assessing domains of memory, perceptual speed, and executive function, in the current population.

Previously, greater total self-reported physical activity (Singh-Manoux et al., 2005; Winneke et al., 2012; Erickson et al., 2013) or exercise (Berchicci et al., 2013) levels have been associated with better cognitive function in middle-aged populations. The current investigation did not observe associations between self-reported physical activity and cognitive function for total physical activity or any intensity subset. The current investigation also observed no association between objectively measured physical activity and cognitive function, which is similar to an earlier investigation (Vásquez et al., 2017), utilising similar cognitive measures. In contrast, while Spartano et al. (2019) found no association between MVPA and visual memory or visual perception, they reported an association between a measure of executive function and MVPA. Extending from these previous works (Vásquez et al., 2017; Spartano et al., 2019), we observed no other associations when physical activity was broken into brackets of light-, moderate-, or vigorous-intensity. Taken together, the non-significant results in the current study combined with those of Vásquez et al. (2017) suggest that a cross-sectional relationship between physical activity and cognitive performance is not readily observable in middle-aged adults. It may be that midlife levels of physical activity are more predictive of future cognitive performance, or that the cognitive tasks used are not sensitive enough to detect more subtle effects taking place in middle-age.

There are several potential reasons for the current investigations null findings, and the resulting differences in outcomes from those reported by Spartano et al. (2019). The different cut-points utilized in the investigations between Spartano et al. (2019) and Vásquez et al. (2017) have been raised as a potential reason for the differences observed between their studies (Spartano et al., 2019). However, as the current investigation did not observe independent associations of self-reported or objectively measured physical activity and cognitive performance, it is not likely that the different measurement methods were the main reasons for different findings. Sample population differences including variations in age range, education, socioeconomic status, and activity levels, may have contributed to the different outcomes observed. Several of the discussed investigations (Singh-Manoux et al., 2005; Winneke et al., 2012; Berchicci et al., 2013; Erickson et al., 2013; Spartano et al., 2019) exhibited a broader age and a larger population cohort, while the current cohort was relatively homogenous in age, education, and socioeconomic status. Compared to the cohort described by Spartano et al. who completed an average of 27.7 (± 20.9) min MVPA/day, the cohort in the current study completed ~112 (± 81) min MVPA/day, with the former research concluding that 10–21 min MVPA per day was beneficial to executive function. As this latter level sits close to the recommended weekly physical activity level, it can be considered high and may not be reflective of the wider population. Although the current investigation utilised quartiles, due to the high levels of physical activity, the splits of physical activity levels were higher than those of Spartano et al. (2019) which possibly contributed to the differing results. The high levels of physical activity and education, and the lower age range of the current cohort, may have limited our capacity to detect physical activity-cognitive relationships if there is a low activity threshold at which benefits will be observed.

The array of neuropsychological tasks administered may also contribute to the different results obtained across investigations. Firstly, a common issue may arise from task specificity, as cognitive performance is typically characterised into domains, with neuropsychological tests measuring one or more discrete abilities within a domain (Harvey, 2019). This may cause difficulty in disentangling or clarifying cognitive results as several tasks capture aspects of unintended domains (Salthouse, 2005), which may decline at different rates (Hedden and Gabrieli, 2004), or may not appropriately represent the cognitive domains they were designed to measure (Salthouse, 2005). Ceiling and practice effects are often acknowledged when assessing cognitive function in healthy adults (Uttl et al., 2002; Zerr et al., 2018).

Previous literature alluded to the potential importance of light- (Chastin et al., 2019; Spartano et al., 2019) and vigorous-intensity exercise (Brown et al., 2012) on markers of brain health. It has been demonstrated that higher levels of accelerometer-determined light-intensity physical activity was associated with higher total cerebral brain volume in middle-aged adults (Spartano et al., 2019). Higher total brain volume, combined in a composite of brain age, has been associated with cognitive function and decline (Elliott et al., 2019). Additionally, in at-risk middle-aged adults, objectively measured moderate-intensity physical activity was associated with a healthier profile of Alzheimer’s Disease biomarkers (Law et al., 2018). Thus, future cross-sectional investigations of objectively measured physical activity and functional or structural brain imaging may provide more insight into the importance of specific physical activity behaviours in middle-aged adults before developing measurable performance deficits.

The current investigation utilised an objective measure of physical activity in a large cohort of middle-aged adults, however, there are several limitations by which the findings of this study should be considered under. This study investigated healthy, well educated, active middle-aged adults, the results may not be reflective of a wider population group. As this study utilised a cross-sectional design, comment on causal relationships cannot be made. A distinct advantage of the PATH project (Anstey et al., 2012) however, from which this sample is drawn, is its longitudinal design, allowing data from the current sub-study to be utilised in future follow-ups informing on the trajectory of cognitive change. Future data from this cohort may inform on potential protective effects of objective midlife physical activity on late-life cognitive function. The data may also be used to assess possible reverse causality, where associations may be observed due to a decrease in physical activity during the preclinical stages of dementia (Sabia et al., 2017).Considerations to the equipment used must also be given. As the SWA has known limitations when measuring high intensity activity and cannot be submerged in water, the SWA may incorrectly reflect the physical activity of persons conducting such activity. The continued advancements to the sensitivity and accuracy of activity monitors available for research will be essential to understanding the relationship between vigorous physical activity and cognitive health in middle-aged adults. Similarly, advances of computerised cognitive testing in middle-aged cohorts may overcome the sensitivity and ceiling effects cited as potentially impacting the accuracy of results in the current cohort.

Understanding the relationships between physical activity at different intensities and cognitive function in middle-aged adults will help inform physical activity guidelines for healthy ageing. The current study did not demonstrate independent associations of self-reported or objectively measured physical activity and cognitive function in middle-aged adults. Future research in middle-aged cohorts will likely benefit from the increasing availability of validated computerised cognitive assessments (Salthouse, 2000; Finkel et al., 2007; Wild et al., 2008; Bauer et al., 2012; Cherbuin et al., 2015; Santos-Lozano et al., 2017; Schuch et al., 2018; Semkovska et al., 2019; Oyarce et al., 2020). Combining the above methodologies and investigating the associations of objectively measured physical activity during midlife and the longitudinal trajectory of cognitive decline into later adulthood may provide essential insights into the dose of physical activity required to assist healthy cognitive ageing.

## Data availability statement

The datasets presented in this article are not readily available because the PATH study governance arrangements and the commitments made to participants do not allow for unrestricted sharing of research data, we will be happy to make available the analysed data to external parties to verify our findings or for research purposes on a case by case basis after a formal request to, and approval from the PATH Research Committee. Requests to access the datasets should be directed to info@pathstudy.org.au.

## Ethics statement

The studies involving human participants were reviewed and approved by Australian National University Human Research Ethics Committee (Human Ethics Protocol 2016/445). The patients/participants provided their written informed consent to participate in this study.

## Author contributions

CQ contributed to the design, data collection, analysis, and writing of the manuscript. BR, JN, and DP contributed to the design, supervision of data collection, analysis, and writing of the manuscript. NC was the main contributor to the study design and funding, contributed to data analysis, and contributed to the writing and proofing of the manuscript. All authors contributed to the article and approved the submitted version.

## Funding

This research was funded by NHMRC grants 973302, 157125, 1106723. The funders had no role in study design, data analysis, data interpretation, or writing of the report. The corresponding author had full access to all the data in the study and all authors had final responsibility for the decision to submit for publication.

## Conflict of interest

The authors declare that the research was conducted in the absence of any commercial or financial relationships that could be construed as a potential conflict of interest.

## Publisher’s note

All claims expressed in this article are solely those of the authors and do not necessarily represent those of their affiliated organizations, or those of the publisher, the editors and the reviewers. Any product that may be evaluated in this article, or claim that may be made by its manufacturer, is not guaranteed or endorsed by the publisher.
