# Supplementary material for: Physical activity and cognitive function in middle-aged adults: a cross-sectional analysis of the PATH through life study
Source: Front Psychol. 2023 Aug 24;14:1022868. doi: 10.3389/fpsyg.2023.1022868 (PMC10484531; doi:10.3389/fpsyg.2023.1022868)
Supplement: Supplementary file 1 [file Data_Sheet_1.docx]

Table S1a– The association between physical activity, measured by the Physical Activity Recall Survey and cognitive function outcomes.

|  |  | Immediate recall |  |  | Delayed recall |  |  | SDMT |  |  | DSBT |  |
| --- | --- | --- | --- | --- | --- | --- | --- | --- | --- | --- | --- | --- |
|  |  | β ± SE | p^#^ |  | β ± SE | p^#^ |  | β ± SE | p^#^ |  | β ± SE | p^#^ |
| Light PA  (min∙week^-1^) | Education* | 0.84 ± 0.36 | 0.07 |  | 0.47 ± 0.36 | 0.47 |  | 0.44 ± 0.37 | 0.74 |  | 0.65 ± 0.37 | 0.44 |
|  | Age | 0.05 ± 0.08 | 0.54 |  | -0.03 ± 0.08 | 0.54 |  | -0.06 ± 0.08 | 0.74 |  | 0.10 ± 0.08 | 0.44 |
|  | Sex^ | 0.36 ± 0.16 | 0.07 |  | 0.39 ± 0.16 | 0.12 |  | -0.11 ± 0.17 | 0.74 |  | 0.03 ± 0.17 | 0.86 |
|  | PAR | -0.05 ± 0.08 | 0.54 |  | -0.04 ± 0.08 | 0.78 |  | -0.06 ± 0.08 | 0.74 |  | 0.05 ± 0.08 | 0.63 |
|  | Intercept | -0.90 ± 0.37 | 0.07 |  | -0.57 ± 0.37 | 0.43 |  | -0.23 ± 0.38 | 0.74 |  | -0.55 ± 0.38 | 0.44 |
|  | Model | F_6,149_=2.4; p =0.03; R^2^=0.05 | |  | F_6,149_=2.2; p =0.05; R^2^=0.04 | |  | F_6,149_=1.1; p =0.38; R^2^=0.003 | |  | F_6,149_=1.0; p =0.44;  R^2^=-0.001 | |
| Moderate PA  ( min∙week^-1^) | Education* | 0.82 ± 0.36 | 0.06 |  | 0.44 ± 0.36 | 0.53 |  | 0.41 ± 0.37 | 0.80 |  | 0.65 ± 0.37 | 0.42 |
|  | Age | 0.05 ± 0.08 | 0.59 |  | -0.03 ± 0.08 | 0.80 |  | -0.06 ± 0.08 | 0.80 |  | 0.10 ± 0.08 | 0.42 |
|  | Sex^ | 0.36 ± 0.16 | 0.06 |  | 0.40 ± 0.16 | 0.11 |  | -0.10 0.17 | 0.80 |  | 0.03 ± 0.17 | 0.86 |
|  | PAR | 0.04 ± 0.08 | 0.59 |  | 0.06 ± 0.08 | 0.67 |  | 0.05 ± 0.08 | 0.80 |  | 0.02 ± 0.08 | 0.86 |
|  | Intercept | -0.88 ± 0.37 | 0.06 |  | -0.55 ± 0.37 | 0.48 |  | -0.21 ± 0.38 | 0.80 |  | -0.56 ± 0.38 | 0.42 |
|  | Model | F_6,149_=2.4; p =0.03; R^2^=0.05 | |  | F_6,149_=2.2; p =0.04; R^2^=0.05 | |  | F_6,149_=1.1; p =0.39; R^2^=0.002 | |  | F_6,149_=0.92; p =0.48; R^2^=-0.003 | |
| Vigorous PA  ( min∙week^-1^) | Education* | 0.83 ± 0.36 | 0.08 |  | 0.46 ± 0.36 | 0.48 |  | 0.42 ± 0.37 | 0.80 |  | 0.66 ± 0.37 | 0.42 |
|  | Age | 0.05 ± 0.08 | 0.66 |  | -0.04 ± 0.08 | 0.80 |  | -0.05 ± 0.08 | 0.80 |  | 0.10 ± 0.08 | 0.42 |
|  | Sex^ | 0.35 ± 0.16 | 0.09 |  | 0.38 ± 0.17 | 0.16 |  | -0.08 ± 0.17 | 0.80 |  | 0.02 ± 0.17 | 0.89 |
|  | PAR | -0.04 ± 0.08 | 0.66 |  | -0.03 ± 0.08 | 0.80 |  | 0.10 ± 0.08 | 0.80 |  | -0.01 ± 0.08 | 0.89 |
|  | Intercept | -0.88 ± 0.37 | 0.08 |  | -0.55 ± 0.37 | 0.47 |  | -0.24 ± 0.37 | 0.80 |  | -0.56 ± 0.38 | 0.42 |
|  | Model | F_6,149_=2.4; p =0.03; R^2^=0.05 | |  | F_6,149_=2.2; p =0.049; R^2^=0.04 | |  | F_6,149_=1.2; p =0.30; R^2^=0.01 | |  | F_6,149_=0.9; p =0.49; R^2^=-0.003 | |
| MVPA  ( min∙week^-1^) | Education* | 0.83 ± 0.36 | 0.07 |  | 0.45 ± 0.36 | 0.49 |  | 0.41 ± 0.37 | 0.83 |  | 0.66 ± 0.37 | 0.42 |
|  | Age | 0.05 ± 0.08 | 0.63 |  | -0.03 ± 0.08 | 0.92 |  | -0.05 ± 0.08 | 0.83 |  | 0.10 ± 0.17 | 0.42 |
|  | Sex^ | 0.36 ± 0.16 | 0.07 |  | 0.40 ± 0.17 | 0.12 |  | -0.08 ± 0.17 | 0.83 |  | 0.03 ± 0.17 | 0.98 |
|  | PAR | -0.001 ± 0.08 | 0.99 |  | 0.01 ± 0.08 | 0.92 |  | 0.10 ± 0.08 | 0.83 |  | 0.002 ± 0.08 | 0.98 |
|  | Intercept | -0.88 ± 0.37 | 0.07 |  | -0.56 ± 0.37 | 0.45 |  | -0.23 ± 0.37 | 0.83 |  | -0.57 ± 0.38 | 0.42 |
|  | Model | F_6,149_=2.4; p =0.03; R^2^=0.05 | |  | F_6,149_=2.1; p =0.05; R^2^=0.04 | |  | F_6,149_=1.2; p =0.29; R^2^=0.01 | |  | F_6,149_=0.9; p =0.49; R^2^=-0.003 | |
| TOTAL PA  (MET:mins∙week^-1^) | Education* | 0.84 ± 0.36 | 0.08 |  | 0.46 ± 0.36 | 0.48 |  | 0.42 ± 0.37 | 0.80 |  | 0.65 ± 0.37 | 0.42 |
|  | Age | 0.05 ± 0.08 | 0.65 |  | -0.04 ± 0.08 | 0.56 |  | -0.06 ± 0.08 | 0.80 |  | 0.10 ± 0.08 | 0.42 |
|  | Sex^ | 0.35 ± 0.16 | 0.08 |  | 0.39 ± 0.16 | 0.14 |  | -0.09 ± 0.17 | 0.80 |  | 0.03 ± 0.17 | 0.85 |
|  | PAR | -0.03 ± 0.08 | 0.68 |  | -002 ± 0.08 | 0.90 |  | 0.06 ± 0.08 | 0.80 |  | 0.02 ± 0.08 | 0.85 |
|  | Intercept | -0.88 ± 0.37 | 0.08 |  | -0.56 ± 0.37 | 0.6 |  | -0.22 ± 0.37 | 0.80 |  | -0.57 ± 0.38 | 0.42 |
|  | Model | F_6,149_=2.4; p =0.03; R^2^=0.05 | |  | F_6,149_=2.1; p =0.05; R^2^=0.04 | |  | F_6,149_=1.1; p =0.39; R^2^=0.002 | |  | F_6,149_=0.9; p =0.48; R^2^=-0.003 | |
| Notes: Model: age, sex, education. ^#^ adjusted for multiple comparisons; ^ compared to men; high school certificate compared to university. PAR: Physical Activity Recall Survey; SDMT: Symbol Digit Modalities Test; DSBT: Digit-Span Backwards Task; PA: Physical activity; MVPA; moderate-to-vigorous physical activity. β are standardized. | | | | | | | | | | | | |

Table S1b – The association between physical activity, measured by the SenseWear Armband™ and cognitive function outcomes.

|  |  | Immediate recall |  |  | Delayed recall |  |  | SDMT |  |  | DSBT |  |
| --- | --- | --- | --- | --- | --- | --- | --- | --- | --- | --- | --- | --- |
|  |  | β ± SE | p^#^ |  | β ± SE | p^#^ |  | β ± SE | p^#^ |  | β ± SE | p^#^ |
| Light PA  (min∙week^-1^) | Education* | 0.82 ±0.36 | 0.08 |  | 0.42 ± 0.37 | 0.58 |  | 0.44 ± 0.37 | 0.88 |  | 0.66 ± 0.37 | 0.42 |
|  | Age | 0.05 ± 0.08 | 0.60 |  | -0.02 ± 0.08 | 0.89 |  | -0.07 ± 0.08 | 0.88 |  | 0.10 ± 0.08 | 0.42 |
|  | Sex^ | 0.35 ± 0.16 | 0.08 |  | 0.38 ± 0.16 | 0.15 |  | -0.11 ± 0.17 | 0.88 |  | 0.03 ± 0.17 | 0.94 |
|  | SWA | 0.02 ± 0.08 | 0.78 |  | 0.07 ± 0.08 | 0.58 |  | -0.02 ± 0.08 | 0.88 |  | -0.01 ± 0.08 | 0.94 |
|  | Intercept | -0.87 ± 0.37 | 0.08 |  | 0.42 ± 0.36 | 0.58 |  | -0.23 ± 0.38 | 0.88 |  | -0.57 ± 0.38 | 0.42 |
|  | Model | F_6,149_=2.4; p =0.03; R^2^=0.05 | |  | F_6,149_=2.3; p =0.04; R^2^=0.05 | |  | F_6,149_=1.00; p =0.43; R^2^=-0.0003 | |  | F_6,149_=0.9; p =0.49; R^2^=-0.0003 | |
| Moderate PA  ( min∙week^-1^) | Education* | 0.83 ± 0.36 | 0.08 |  | 0.46 ± 0.36 | 0.48 |  | 0.41 ± 0.37 | 0.87 |  | 0.63 ± 0.37 | 0.38 |
|  | Age | 0.05 ± 0.08 | 0.63 |  | -0.03 ± 0.08 | 0.92 |  | -0.06 ± 0.18 | 0.87 |  | 0.10 ± 0.08 | 0.38 |
|  | Sex^ | 0.37 ± 0.17 | 0.08 |  | 0.39 ± 0.17 | 0.19 |  | -0.06 ± 0.18 | 0.87 |  | 0.09 ± 0.18 | 0.62 |
|  | SWA | 0.01 ± 0.08 | 0.89 |  | -0.01 ± 0.08 | 0.92 |  | 0.07 ± 0.09 | 0.87 |  | 0.08 ± 0.09 | 0.38 |
|  | Intercept | -0.88 ± 0.37 | 0.08 |  | -0.56 ± 0.37 | 0.46 |  | -0.23 ± 0.37 | 0.87 |  | -0.58 ± 0.37 | 0.34 |
|  | Model | F_6,149_=2.4; p =0.03; R^2^=0.05 | |  | F_6,149_=2.1; p =0.05; R^2^=0.08 | |  | F_6,149_=1.1; p =0.36; R^2^=0.004 | |  | F_6,149_=1.1; p =0.38; R^2^=0.003 | |
| Vigorous PA  ( min∙week^-1^) | Education* | 0.81 ± 0.36 | 0.06 |  | 0.44 ± 0.36 | 0.52 |  | 0.41 ± 0.37 | 0.82 |  | 0.64 ± 0.37 | 0.41 |
|  | Age | 0.06 ± 0.08 | 0.47 |  | -0.03 ± 0.08 | 0.84 |  | -0.06 ± 0.08 | 0.82 |  | 0.11 ± 0.08 | 0.41 |
|  | Sex^ | 0.40 ± 0.17 | 0.06 |  | 0.42 ± 0.17 | 0.09 |  | -0.07 ± 0.17 | 0.82 |  | 0.06 ± 0.17 | 0.73 |
|  | SWA | 0.09 ± 0.08 | 0.33 |  | 0.06 ± 0.08 | 0.66 |  | 0.08 ± 0.09 | 0.82 |  | 0.06 ± 0.09 | 0.56 |
|  | Intercept | -0.91 ± 0.37 | 0.06 |  | -0.57 ± 0.37 | 0.42 |  | -0.24 ± 0.37 | 0.82 |  | -0.58 ± 0.38 | 0.41 |
|  | Model | F_6,149_=2.6; p =0.02; R^2^=0.06 | |  | F_6,149_=2.2; p =0.04; R^2^=0.05 | |  | F_6,149_=1.1; p =0.34; R^2^=0.005 | |  | F_6,149_=1; p =0.42; R^2^=<0.001 | |
| MVPA  ( min∙week^-1^) | Education* | 0.82 ± 0.36 | 0.07 |  | 0.46 ± 0.36 | 0.49 |  | 0.40 ± 0.37 | 0.92 |  | 0.63 ± 0.37 | 0.37 |
|  | Age | 0.05 ± 0.08 | 0.62 |  | -0.03 ± 0.08 | 0.94 |  | -0.06 ± 0.08 | 0.92 |  | 0.11 ± 0.08 | 0.37 |
|  | Sex^ | 0.38 ± 0.17 | 0.07 |  | 0.40 ± 0.17 | 0.18 |  | -0.05 ± 0.18 | 0.92 |  | 0.10 ± 0.18 | 0.59 |
|  | SWA | 0.03 ± 0.08 | 0.74 |  | 0.003 ± 0.08 | 0.96 |  | 0.08 ± 0.09 | 0.92 |  | 0.09 ± 0.09 | 0.37 |
|  | Intercept | -0.89 ± 0.37 | 0.07 |  | -0.56 ± 0.37 | 0.46 |  | -0.23 ± 0.37 | 0.92 |  | -0.58 ± 0.37 | 0.37 |
|  | Model | F_6,149_=2.4; p =0.03; R^2^=0.05 | |  | F_6,149_=2.4; p =0.03; R^2^=0.05 | |  | F_6,149_=1.1; p =0.34; R^2^=0.01 | |  | F_6,149_=1.1; p =0.36; R^2^=0.004 | |
| TOTAL PA  (MET:mins∙week^-1^) | Education* | 0.80 ± 0.36 | 0.07 |  | 0.43 ± 0.36 | 0.55 |  | 0.39 ± 0.37 | 0.88 |  | 0.60 ± 0.37 | 0.38 |
|  | Age | 0.05 ± 0.08 | 0.51 |  | -0.03 ± 0.08 | 0.82 |  | -0.06 ± 0.08 | 0.88 |  | 0.11 ± 0.08 | 0.38 |
|  | Sex^ | 0.39 ± 0.17 | 0.07 |  | 0.42 ± 0.17 | 0.10 |  | -0.06 ± 0.17 | 0.88 |  | 0.09 ± 0.17 | 0.38 |
|  | SWA | 0.05 ± 0.08 | 0.51 |  | 0.05 ± 0.08 | 0.81 |  | 0.08 ± 0.09 | 0.88 |  | 0.10 ± 0.08 | 0.38 |
|  | Intercept | -0.88 ± 0.37 | 0.07 |  | -0.56 ± 0.37 | 0.46 |  | -0.22 ± 0.37 | 0.88 |  | -0.56 ± 0.37 | 0.38 |
|  | Model | F_6,149_=2.4; p =0.03; R^2^=0.05 | |  | F_6,149_=2.2; p =0.047; R^2^=0.04 | |  | F_6,149_=1.1; p =0.34; R^2^=0.01 | |  | F_6,149_=1.2; p =0.33; R^2^=0.01 | |
| Notes: Model: age, sex, education. ^#^ adjusted for multiple comparisons; ^ compared to men; high school certificate compared to university. SWA: SenseWear Armband^TM^; SDMT: Symbol Digit Modalities Test; DSBT: Digit-Span Backwards Task; PA: Physical activity; MVPA; moderate-to-vigorous physical activity. β are standardised. | | | | | | | | | | | | |

Table S2a – The association between physical activity, measured by the Physical Activity Recall questionnaire and cognitive function when controlling for age, sex, education, BMI, hypertension, diabetes, and audit class.

|  |  | Immediate recall |  | |  | Delayed recall | |  |  | SDMT | |  |  | DSBT |  | |
| --- | --- | --- | --- | --- | --- | --- | --- | --- | --- | --- | --- | --- | --- | --- | --- | --- |
|  |  | β ± SE | p# | |  | β ± SE | | p# |  | β ± SE | | p# |  | β ± SE | p# | |
| Light PA (min∙week-1) | BMI | -0.11 ± 0.09 | 0.51 | |  | -0.11 ± 0.09 | | 0.79 |  | -0.01 ± 0.09 | | 0.88 |  | 0.01 ± 0.09 | 0.98 | |
|  | Hypertension | -0.17 ± 0.39 | 0.77 | |  | -0.002 ± 0.39 | | 0.99 |  | -0.56 ± 0.40 | | 0.65 |  | 0.50 ± 0.40 | 0.89 | |
|  | Audit class^Y^ | -0.99 ± 0.84 | 0.51 | |  | -0.19 ± 0.85 | | 0.88 |  | 0.95 ± 0.87 | | 0.65 |  | 0.51 ± 0.54 | 0.89 | |
|  | Diabetes^+^ | 0.66 ± 0.71 | 0.86 | |  | 0.72 ± 0.72 | | 0.88 |  | 0.40 ± 0.74 | | 0.75 |  | -0.98 ± 0.73 | 0.89 | |
|  | Education* | 0.80 ± 0.37 | 0.29 | |  | 0.42 ± 0.37 | | 0.79 |  | 0.52 ± 0.38 | | 0.65 |  | 0.58 ± 0.38 | 0.89 | |
|  | Age | 0.07 ± 0.08 | 0.56 | |  | -0.03 ± 0.08 | | 0.88 |  | -0.07 ± 0.08 | | 0.72 |  | 0.08 ± 0.08 | 0.89 | |
|  | Sex^ | 0.36 ± 0.17 | 0.29 | |  | 0.42 ± 0.18 | | 0.24 |  | -0.07 ± 0.18 | | 0.75 |  | -0.01 ± 0.18 | 0.98 | |
|  | PAR | -0.07 ± 0.08 | 0.56 | |  | -0.06 ± 0.08 | | 0.88 |  | -0.06 ± 0.08 | | 0.72 |  | 0.04 ± 0.08 | 0.89 | |
|  | Intercept | -1.21 ± 1.02 | 0.51 | |  | -1.44 ± 1.03 | | 0.79 |  | -0.71 ± 1.06 | | 0.75 |  | 0.30 ± 1.04 | 0.96 | |
|  | Model | F_14,141_=1.5; p =0.10;  R2=0.05, ∆ R^2^ = 0 | | |  | F_14,141_=1.3; p =0.20;  R2=0.03, ∆ R^2^ = -0.02 | | |  | F_14,141_=0.74; p =0.73;  R2=-0.02, ∆ R^2^ = 0.022 | | |  | F_14,141_=0.74; p =0.39;  R2=0.007, ∆ R^2^ = 0.006 | | |
| Moderate PA  ( min∙week-1) | BMI | -0.10 ± 0.09 | 0.57 | |  | -0.10 ± 0.09 | | 0.85 |  | 0.002 ± 0.09 | | 0.98 |  | 0.01 ± 0.09 | 0.96 | |
|  | Hypertension | -0.18 ± 0.39 | 0.80 | |  | -0.02 ± 0.39 | | 0.96 |  | -0.58 ± 0.40 | | 0.71 |  | 0.50 ± 0.40 | 0.90 | |
|  | Audit class^Y^ | -1.02 ± 0.84 | 0.57 | |  | -0.22 ± 0.85 | | 0.90 |  | 0.91 ± 0.87 | | 0.71 |  | 0.53 ± 0.86 | 0.90 | |
|  | Diabetes^+^ | 0.63 ± 0.71 | 0.60 | |  | 0.70 ± 0.72 | | 0.85 |  | 0.37 ± 0.74 | | 0.80 |  | -0.97 ± 0.73 | 0.90 | |
|  | Education* | 0.78 ± 0.37 | 0.27 | |  | 0.39 ± 0.37 | | 0.85 |  | 0.49 ± 0.38 | | 0.71 |  | 0.58 ± 0.38 | 0.90 | |
|  | Age | 0.07 ± 0.08 | 0.60 | |  | -0.02 ± 0.08 | | 0.90 |  | -0.07 ± 0.08 | | 0.80 |  | 0.08 ± 0.08 | 0.90 | |
|  | Sex^ | 0.37 ± 0.18 | 0.27 | |  | 0.43 ± 0.18 | | 0.24 |  | -0.06 ± 0.18 | | 0.80 |  | -0.01 ± 0.18 | 0.96 | |
|  | PAR | 0.03 ± 0.08 | 0.84 | |  | 0.04 ± 0.08 | | 0.90 |  | 0.05 ± 0.08 | | 0.80 |  | 0.02 ± 0.08 | 0.96 | |
|  | Intercept | -1.14 ± 1.02 | 0.57 | |  | -1.36 ± 1.03 | | 0.85 |  | -0.62 ± 1.06 | | 0.80 |  | 0.28 ± 1.04 | 0.96 | |
|  | Model | F_14,141_=1.5; p =0.12;  R2=0.04, ∆ R^2^ = -0.01 | | |  | F_14,141_=1.3; p =0.22;  R2=0.03, ∆ R^2^ = -0.01 | | |  | F_14,141_=0.7; p =0.75;  R2=-0.03, ∆ R^2^ = -0.04 | | |  | F_14,141_=1.1; p =0.40;  R2=0.01, ∆ R^2^ = 0.007 | | |
| Vigorous PA  ( min∙week-1) | BMI | -0.11 ± 0.09 | 0.56 | |  | -0.11 ± 0.09 | | 0.85 |  | 0.01 ± 0.09 | | 0.94 |  | 00.01 ± 0.09 | 0.95 | |
|  | Hypertension | -0.17 ± 0.39 | 0.82 | |  | -0.004 ± **0.40** | | 0.99 |  | -0.61 ± 0.40 | | 0.72 |  | 0.52 ± 0.40 | 0.92 | |
|  | Audit class^Y^ | 0.79 ± 0.37 | 0.56 | |  | -0.16 ± z0.86 | | 0.91 |  | 0.76 ± 0.88 | | 0.72 |  | 0.58 ± 0.86 | 0.92 | |
|  | Diabetes^+^ | 0.63 ± 0.72 | 0.64 | |  | 0.69 ± 0.72 | | 0.86 |  | 0.43 ± 0.38 | | 0.77 |  | -0.98 ± 0.73 | 0.92 | |
|  | Education* | 0.79 ± 0.37 | 0.37 | |  | 0.41 ± 0.37 | | 0.86 |  | 0.49 ± 0.38 | | 0.72 |  | 0.59 ± 0.38 | 0.92 | |
|  | Age | 0.06 ± 0.08 | 0.65 | |  | -0.03 ± 0.08 | | 0.91 |  | -0.05 ± 0.08 | | 0.77 |  | 0.08 ± 0.08 | 0.92 | |
|  | Sex^ | 0.35 ± 0.18 | 0.37 | |  | 0.41 ± 0.18 | | 0.36 |  | -0.03 ± 0.18 | | 0.94 |  | -0.03 ± 0.18 | 0.95 | |
|  | PAR | -0.03 ± 0.08 | 0.85 | |  | -0.04 ± 0.08 | | 0.91 |  | 0.10 ± 0.09 | | 0.72 |  | -0.03 ± 0.08 | 0.95 | |
|  | Intercept | -1.16 ± 1.02 | 0.56 | |  | -1.9 ± 1.03 | | 0.85 |  | -0.67 ± 1.05 | | 0.77 |  | 0.27 ± 1.04 | 0.95 | |
|  | Model | F_14,141_=1.5; p =0.12;  R2=0.04, ∆ R^2^ = -0.01 | | |  | F_14,141_=1.3; p =0.22;  R2=0.03, ∆ R^2^ = -0.02 | | |  | F_14,141_=0.3=8; p =0.67;  R2=-0.02, ∆ R^2^ = 0.03 | | |  | F_14,141_=1.1=8; p =0.39;  R2=0.01, ∆ R^2^ = -0.007 | | |
| MVPA  ( min∙week-1) | BMI | -0.11 ± 0.09 | 0.55 | |  | -0.11 ± 0.09 | | 0.82 |  | 0.01 ± 0.09 | | 0.88 |  | 0.01 ± 0.09 | 0.94 | |
|  | Hypertension | -0.18 ± 0.39 | 0.82 | |  | -0.01 ± 0.40 | | 0.97 |  | -0.60 ± 0.40 | | 0.68 |  | 0.51 ± 0.40 | 0.92 | |
|  | Audit class^Y^ | -1.02 ± 0.84 | 0.55 | |  | -0.22 ± 0.85 | | 0.94 |  | 0.80 ± 0.87 | | 0.68 |  | 0.55 ± 0.86 | 0.92 | |
|  | Diabetes^+^ | 0.64 ± 0.71 | 0.61 | |  | 0.71 ± 0.72 | | 0.82 |  | 0.40 ± 0.74 | | 0.80 |  | -0.97 ± 0.73 | 0.92 | |
|  | Education* | 0.79 ± 0.37 | 0.31 | |  | 0.40 ± 0.37 | | 0.82 |  | 0.48 ± 0.38 | | 0.68 |  | 0.59 ± 0.38 | 0.92 | |
|  | Age | 0.07 ± 0.08 | 0.61 | |  | -0.03 ± 0.08 | | 0.94 |  | -0.06 ± 0.08 | | 0.80 |  | 0.08 ± 0.08 | 0.92 | |
|  | Sex^ | 0.37 ± 0.18 | 0.31 | |  | 0.43 ± 0.18 | | 0.58 |  | -0.03 ± 0.18 | | 0.88 |  | -0.02 ± 0.18 | 0.94 | |
|  | PAR | -0.003 ± 0.08 | 0.97 | |  | -0.003 ± 0.08 | | 0.97 |  | 0.10 ± 0.09 | | 0.68 |  | -0.01 ± 0.08 | 0.94 | |
|  | Intercept | -1.16 ± 1.02 | 0.55 | |  | -1.39 ± 1.03 | | 0.82 |  | -0.62 ± 1.05 | | 0.80 |  | 0.26 ± 1.04 | 0.94 | |
|  | Model | F_14,141_=1.5; p =0.12;  R2=0.04, ∆ R^2^ = -0.01 | | |  | F_14,141_=1.3; p =0.23;  R2=0.02, ∆ R^2^ = -0.02 | | |  | F_14,141_=0.8; p =0.66;  R2=-0.02, ∆ R^2^ = -0.03 | | |  | F_14,141_=1.1; p =0.40;  R2=0.005, ∆ R^2^ = 0.02 | | |
| TOTAL PA (MET:mins∙week^-1^) | BMI | -0.11 ± 0.09 | | 0.54 |  | -0.11 ± 0.09 | 0.83 | |  | 0.002 ± 0.09 | 0.97 | |  | 0.01 ± 0.09 | | 0.99 |
|  | Hypertension | -0.17 ± 0.39 | | 0.77 |  | -0.002 ± 0.40 | 0.90 | |  | -0.59 ± 0.41 | 0.74 | |  | 0.51 ± 0.40 | | 0.91 |
|  | Audit class^Y^ | -0.97 ±0.85 | | 0.54 |  | -0.17 ±0.85 | 0.90 | |  | 0.84 ± 0.88 | 0.74 | |  | 0.53 ± 0.86 | | 0.91 |
|  | Diabetes^+^ | 0.63 ± 0.71 | | 0.63 |  | 0.70 ± 0.72 | 0.83 | |  | 0.39 ± 0.74 | 0.82 | |  | -0.97 ± 0.73 | | 0.91 |
|  | Education* | 0.80 ± 0.37 | | 0.36 |  | 0.41 ± 0.37 | 0.83 | |  | 0.49 ± 0.38 | 0.74 | |  | 0.59 ± 0.38 | | 0.91 |
|  | Age | 0.06 ± 0.08 | | 0.65 |  | -0.03 ± 0.08 | 0.90 | |  | -0.06 ± 0.08 | 0.82 | |  | 0.08 ± 0.08 | | 0.91 |
|  | Sex^ | 0.35 ± 0.18 | | 0.36 |  | 0.41 ± 0.18 | 0.33 | |  | -0.05 ± 0.18 | 0.83 | |  | -0.01 ± 0.18 | | 0.99 |
|  | PAR | -0.04 ± 0.08 | | 0.77 |  | -0.04 ± 0.08 | 0.90 | |  | 0.06 ± 0.09 | 0.82 | |  | 0.001 ± 0.08 | | 0.99 |
|  | Intercept | -1.18 ± 1.02 | | 0.54 |  | -1.41 ± 1.03 | 0.83 | |  | -0.63 ± 1.06 | 0.82 | |  | 0.27 ± 1.04 | | 0.99 |
|  | Model | F_14,141_=1.5; p =0.12;  R2=0.04, ∆ R^2^ = -0.01 | | |  | F_14,141_=1.3; p =0.22;  R2=0.03, ∆ R^2^ = -0.01 | | |  | F_14,141_=0.7; p =0.74;  R2=-0.03, ∆ R^2^ = -0.032 | | |  | F_14,141_=1.1; p =0.41;  R2=0.005, ∆ R^2^ = 0.002 | | |
| Notes: Model: age, sex, education, BMI, hypertension, diabetes, and audit class. ^#^ adjusted for multiple comparisons; ^ compared to men; high school certificate compared to university; ^+^ compared to those with diabetes; ^Y^heavy drinkers compared to abstainers. SDMT: Symbol Digit Modalities Test; DSBT: Digit-Span Backwards Task; PA: Physical activity; MVPA; moderate-to-vigorous physical activity. β are standardised. ∆ R^2^ compared to model presented in Table S1a | | | | | | | | | | | | | | | | |

Table S2b – The association between physical activity, measured by the SenseWear Armband™ and cognitive function when controlling for age, sex, education, BMI, hypertension, diabetes, and audit class.

|  |  | Immediate recall |  | |  | Delayed recall | |  |  | SDMT | |  |  | DSBT | |  |
| --- | --- | --- | --- | --- | --- | --- | --- | --- | --- | --- | --- | --- | --- | --- | --- | --- |
|  |  | β ± SE | p# | |  | β ± SE | | p# |  | β ± SE | | p# |  | β ± SE | | p# |
| Light PA (min∙week-1) | BMI | -0.12 ± 0.10 | 0.53 | |  | -0.10 ± 0.10 | | 0.85 |  | -0.03 ± 0.10 | | 0.79 |  | 0.004 ± 0.10 | | 0.97 |
|  | Hypertension | -0.18 ± 0.39 | 0.85 | |  | -0.02 ± 0.39 | | 0.97 |  | -0.57 ± 0.41 | | 0.64 |  | 0.51 ± 0.40 | | 0.91 |
|  | Audit class^Y^ | -1.01 ± 0.84 | 0.61 | |  | -0.23 ± 0.85 | | 0.91 |  | 0.94 ± 0.87 | | 0.79 |  | 0.54 ± 0.86 | | 0.93 |
|  | Diabetes^+^ | 0.65 ± 0.71 | 0.61 | |  | 0.70 ± 0.72 | | 0.85 |  | 0.39 ± 0.74 | | 0.64 |  | -0.96 ± 0.73 | | 0.91 |
|  | Education* | 0.80 ± 0.37 | 0.27 | |  | 0.39 ± 0.43 | | 0.91 |  | 0.52 ± 0.39 | | 0.64 |  | 0.59 ± 0.38 | | 0.91 |
|  | Age | 0.06 ± 0.08 | 0.66 | |  | -0.02 ± 0.08 | | 0.91 |  | -0.07 ± 0.08 | | 0.72 |  | 0.08 ± 0.08 | | 0.97 |
|  | Sex^ | 0.37 ± 0.18 | 0.27 | |  | 0.42 ± 0.18 | | 0.29 |  | -0.06 ± 0.18 | | 0.79 |  | -0.01 ± 0.18 | | 0.97 |
|  | SWA | -0.03 ± 0.09 | 0.90 | |  | 0.02 ± 0.09 | | 0.91 |  | -0.04 ± 0.10 | | 0.79 |  | -0.01 ± 0.09 | | 0.97 |
|  | Intercept | -1.20 ± 1.03 | 0.53 | |  | -1.36 ± 1.04 | | 0.85 |  | -0.72 ± 1.07 | | 0.79 |  | 0.24 ± 1.05 | | 0.97 |
|  | Model | F_14,141_=1.5; p =0.12;  R2=0.04, ∆ R^2^ = 0.01 | | |  | F_14,141_=1.3; p=0.23;  R2=0.02, ∆ R2 = -0.03 | | |  | F_14,141_=0.7; p =0.76;  R2=-0.03, ∆ R^2^ = 0.0297 | | |  | F_14,141_=1.1; p =0.40;  R2=-0.005, ∆ R^2^ = +0.0047 | | |
| Moderate PA  ( min∙week-1) | BMI | -0.10 ± 0.09 | 0.58 | |  | -0.11 ± 0.09 | | 0.82 |  | 0.02 ± 0.09 | | 0.89 |  | 0.03 ± 0.09 | | 0.90 |
|  | Hypertension | -0.18 ± 0.39 | 0.80 | |  | -0.01 ± 0.39 | | 0.97 |  | -0.60 ± 0.40 | | 0.81 |  | 0.50 ± 0.40 | | 0.90 |
|  | Audit class^Y^ | -1.08 ± 0.86 | 0.57 | |  | -0.147 ± 0.87 | | 0.90 |  | 0.77 ± 0.89 | | 0.81 |  | 0.42 ± 0.88 | | 0.90 |
|  | Diabetes^+^ | 0.64 ± 0.71 | 0.59 | |  | 0.71 ± 0.72 | | 0.82 |  | 0.38 ± 0.74 | | 0.83 |  | -0.97 ± 0.73 | | 0.90 |
|  | Education* | 0.78 ± 0.37 | 0.30 | |  | 0.41 ± 0.37 | | 0.82 |  | 0.49 ± 0.38 | | 0.81 |  | 0.58 ± 0.38 | | 0.90 |
|  | Age | 0.07 ± 0.08 | 0.59 | |  | -0.03 ± 0.08 | | 0.90 |  | -0.06 ± 0.08 | | 0.81 |  | 0.09 ± 0.08 | | 0.90 |
|  | Sex^ | 0.38 ± 0.19 | 0.30 | |  | 0.41 ± 0.19 | | 0.44 |  | -0.03 ± 0.19 | | 0.89 |  | 0.02 ± 0.19 | | 0.98 |
|  | SWA | 0.03 ± 0.09 | 0.89 | |  | -0.02 ± 0.09 | | 0.90 |  | 0.07 ± 0.10 | | 0.81 |  | 0.05 ± 0.10 | | 0.90 |
|  | Intercept | -1.15 ± 1.02 | 0.58 | |  | -1.40 ± 1.03 | | 0.82 |  | -0.63 ± 1.06 | | 0.83 |  | 0.29 ± 1.04 | | 0.90 |
|  | Model | F_14,141_=1.5; p =0.12;  R2=0.04, ∆ R^2^ = -0.01 | | |  | F_14,141_=1.3; p=0.23;  R2=0.02, ∆ R^2^ = -0.02 | | |  | F_14,141_=0.7; p =0.74;  R2=-0.02, ∆ R^2^ = -0.024 | | |  | F_14,141_=1.1; p =0.39;  R2=-0.01, ∆ R^2^ = -0.013 | | |
| Vigorous PA  ( min∙week-1) | BMI | -0.08 ± 0.09 | 0.52 | |  | -0.09 ± 0.09 | | 0.81 |  | 0.02 ± 0.09 | | 0.88 |  | 0.03 ± 0.09 | | 0.94 |
|  | Hypertension | -0.16 ± 0.39 | 0.78 | |  | -0.003 ± 0.40 | | 0.99 |  | -0.55 ± 0.40 | | 0.67 |  | 0.52 ± 0.40 | | 0.79 |
|  | Audit class^Y^ | -1.03 ± 0.84 | 0.52 | |  | -0.22 ± 0.85 | | 0.87 |  | 0.91 ± 0.87 | | 0.67 |  | 0.53 ± 0.85 | | 0.81 |
|  | Diabetes^+^ | 0.65 ± 0.71 | 0.52 | |  | 0.71 ± 0.72 | | 0.81 |  | 0.39 ± 0.74 | | 0.81 |  | -0.96 ± 0.73 | | 0.78 |
|  | Education* | 0.78 ± 0.37 | 0.58 | |  | 0.39 ± 0.37 | | 0.81 |  | 0.49 ± 0.38 | | 0.67 |  | 0.57 ± 0.38 | | 0.78 |
|  | Age | 0.07 ± 0.08 | 0.52 | |  | -0.02 ± 0.08 | | 0.87 |  | -0.06 ± 0.08 | | 0.76 |  | 0.09 ± 0.08 | | 0.78 |
|  | Sex^ | 0.41 ± 0.18 | 0.28 | |  | 0.45 ± 0.18 | | 0.22 |  | -0.03 ± 0.19 | | 0.88 |  | 0.02 ± 0.18 | | 0.95 |
|  | SWA | 0.08 ± 0.09 | 0.52 | |  | 0.04 ± 0.09 | | 0.87 |  | 0.08 ± 0.09 | | 0.67 |  | 0.07 ± 0.09 | | 0.79 |
|  | Intercept | -1.20 ± 1.02 | 0.52 | |  | -1.41 ± 1.03 | | 0.81 |  | -0.70 ± 1.06 | | 0.76 |  | 0.23 ± 1.04 | | 0.95 |
|  | Model | F_14,141_=1.5; p =0.10;  R2=0.05, ∆ R^2^ = -0.01 | | |  | F_14,141_=1.3; p=0.22;  R2=0.03, ∆ R^2^ = -0.02 | | |  | F_14,141_=0.8; p =0.71;  R2=-0.02, ∆ R^2^ = -0.025 | | |  | F_14,141_=1.1; p =0.36;  R2=-0.01 ∆ R^2^ = 0.013 | | |
| MVPA  ( min∙week-1) | BMI | -0.09 ± 0.09 | 0.58 | |  | -0.11 ± 0.09 | | 0.82 |  | 0.02 ± 0.10 | | 0.86 |  | 0.03 ± 0.09 | | 0.89 |
|  | Hypertension | -0.18 ± 0.39 | 0.76 | |  | -0.01 ± 0.39 | | 0.97 |  | -0.58 ± 0.40 | | 0.75 |  | 0.51 ± 0.40 | | 0.83 |
|  | Audit class^Y^ | -1.11 ± 0.86 | 0.58 | |  | -0.20 ± 0.87 | | 0.95 |  | 0.75 ± 0.89 | | 0.75 |  | 0.40 ± 0.88 | | 0.89 |
|  | Diabetes^+^ | 0.64 ± 0.71 | 0.58 | |  | 0.71 ± 0.72 | | 0.82 |  | 0.39 ± 0.74 | | 0.82 |  | -0.96 ± 0.73 | | 0.83 |
|  | Education* | 0.78 ± 0.37 | 0.28 | |  | 0.41 ± 0.37 | | 0.82 |  | 0.48 ± 0.38 | | 0.75 |  | 0.57 ± 0.38 | | 0.83 |
|  | Age | 0.07 ± 0.08 | 0.58 | |  | -0.03 ± 0.08 | | 0.95 |  | -0.06 ± 0.08 | | 0.76 |  | 0.09 ± 0.08 | | 0.83 |
|  | Sex^ | 0.40 ± 0.19 | 0.28 | |  | 0.42 ± 0.19 | | 0.43 |  | -0.01 ± 0.19 | | 0.95 |  | 0.03 ± 0.19 | | 0.92 |
|  | SWA | 0.04 ± 0.10 | 0.76 | |  | -0.01 ± 0.10 | | 0.97 |  | 0.08 ± 0.10 | | 0.75 |  | 0.07 ± 0.10 | | 0.83 |
|  | Intercept | -1.15 ± 1.02 | 0.58 | |  | -1.39 ± 1.03 | | 0.82 |  | -0.63 ± 1.06 | | 0.82 |  | 0.29 ± 1.04 | | 0.90 |
|  | Model | F_14,141_=1.5; p =0.12;  R2=0.04, ∆ R^2^ = -0.01 | | |  | F_14,141_=1.3; p=0.23;  R2=0.02, ∆ R^2^ = -0.03 | | |  | F_14,141_=0.7; p =0.72;  R2=-0.02, ∆ R^2^ = -0.03 | | |  | F_14,141_=1.1; p =0.37;  R2=-0.008, ∆ R^2^ = <0.001 | | |
| TOTAL PA (MET:mins∙week^-1^) | BMI | -0.08 ± 0.10 | | 0.64 |  | -0.10 ± 0.10 | 0.84 | |  | 0.04 ± 0.10 | 0.85 | |  | 0.06 ±0.10 | 0.81 | |
|  | Hypertension | -0.18 ± 0.39 | | 0.74 |  | -0.01 ± 0.39 | 0.97 | |  | -0.57 ± 0.40 | 0.73 | |  | 0.51 ± 0.40 | 0.78 | |
|  | Audit class^Y^ | -1.12 ± 0.85 | | 0.61 |  | -0.26 ± 0.86 | 0.91 | |  | 0.77 ± 0.88 | 0.73 | |  | 0.36 ± 0.87 | 0.85 | |
|  | Diabetes^+^ | 0.64 ± 0.71 | | 0.62 |  | 0.71 ± 0.72 | 0.84 | |  | 0.39 ± 0.74 | 0.8 | |  | -0.96 ± 0.73 | 0.78 | |
|  | Education* | 0.77 ± 0.37 | | 0.30 |  | 0.39 ± 0.38 | 0.84 | |  | 0.47 ± 0.38 | 0.73 | |  | 0.55 ± 0.38 | 0.78 | |
|  | Age | 0.07 ± 0.08 | | 0.62 |  | -0.02 ± 0.08 | 0.91 | |  | -0.06 ± 0.08 | 0.81 | |  | 0.09 ±0.08 | 0.78 | |
|  | Sex^ | 0.40 ± 0.18 | | 0.30 |  | 0.44 ± 0.19 | 0.29 | |  | -0.02 ± 0.19 | 0.91 | |  | 0.05 ± 0.19 | 0.87 | |
|  | SWA | 0.06 ± 0.10 | | 0.74 |  | 0.02 ± 0.10 | 0.91 | |  | 0.09 ± 0.10 | 0.73 | |  | 0.10 ± 0.10 | 0.78 | |
|  | Intercept | -1.12 ± 1.02 | | 0.61 |  | -1.38 ± 1.03 | 0.84 | |  | -0.60 ± 1.06 | 0.82 | |  | 0.33 ± 1.04 | 0.87 | |
|  | Model | F_14,141_=1.5; p =0.12;  R2=0.04, ∆ R^2^ = -0.01 | | |  | F_14,141_=1.3; p =0.23;  R2=0.04, ∆ R^2^ = 0.00 | | |  | F_14,141_=0.8; p =0.72;  R2=-0.02, ∆ R^2^ = -0.03 | | |  | F_14,141_=1.1; p =0.33;  R2=0.01, ∆ R^2^ = 0.00 | | |
| Notes: Model: age, sex, education, BMI, hypertension, diabetes, and audit class. ^#^ adjusted for multiple comparisons; ^ compared to men; high school certificate compared to university; ^+^ compared to those with diabetes; ^Y^heavy drinkers compared to abstainers. SDMT: Symbol Digit Modalities Test; DSBT: Digit-Span Backwards Task; PA: Physical activity; MVPA; moderate-to-vigorous physical activity. β are standardised. ∆ R^2^ compared to model presented in Table S1b | | | | | | | | | | | | | | | | |

Table S3a – The association between physical activity, measured by the Physical Activity Recall survey, and memory and fluid intelligence.

|  |  | Memory | |  |  | Fluid intelligence | |  |  |
| --- | --- | --- | --- | --- | --- | --- | --- | --- | --- |
|  | Variable | β ± SE | | p# |  | β ± SE | | p# |  |
| Light PA (min∙week^-1^) | Education* | 0.65 ± 0.35 | | 0.15 |  | 0.60 ± 0.27 | | 0.14 |  |
|  | Age | 0.01 ± 0.16 | | 0.92 |  | 0.01 ± 0.06 | | 0.82 |  |
|  | Sex^ | 0.37 ± 0.16 | | 0.13 |  | 0.17 ± 0.12 | | 0.39 |  |
|  | PAR | -0.05 ± 0.08 | | 0.64 |  | -0.03 ± 0.06 | | 0.77 |  |
|  | Intercept | -0.73 ± 0035 | | 0.14 |  | -0.56 ± 0.27 | | 0.14 |  |
|  | Model | F_6,149_=2.3; p =0.04;  R2=0.05 | | |  | F6_,149_=2.0; p =0.07;  R2=0.04 | | |  |
| Moderate PA ( min∙week^-1^) | Education* | 0.63 ± 0.35 | | 0.17 |  | 0.58 ± 0.27 | | 0.16 |  |
|  | Age | 0.01 ± 0.08 | | 0.91 |  | 0.01 ± 0.06 | | 0.80 |  |
|  | Sex^ | 0.38 ± 0.16 | | 0.12 |  | 0.17 ± 0.12 | | 0.37 |  |
|  | PAR | 0.05 ± 0.08 | | 0.61 |  | 0.04 ± 0.06 | | 0.55 |  |
|  | Intercept | -0.71 ± 0.35 | | 0.16 |  | -0.55 ± 0.27 | | 0.16 |  |
|  | Model | F_6,149_=2.3; p =0.04;  R2=0.05 | | |  | F_6,149_=2.1; p =0.06;  R2=0.04 | | |  |
| Vigorous PA ( min∙week^-1^) | Education* | 0.65 ± 0.35 | | 0.15 |  | 0.59 ± 0.27 | | 0.15 |  |
|  | Age | 0.004 ± 0.08 | | 0.96 |  | 0.01 ± 0.06 | | 0.94 |  |
|  | Sex^ | 0.36 ± 0.16 | | 0.15 |  | 0.17 ± 0.12 | | 0.40 |  |
|  | PAR | -0.03 ± 0.08 | | 0.77 |  | 0.004 ± 0.06 | | 0.94 |  |
|  | Intercept | -0.71 ± 0.35 | | 0.15 |  | -0.56 ± 0.27 | | 0.15 |  |
|  | Model | F_6,149_=2.2; p =0.04;  R2=0.05 | | |  | F_6,149_=2.0; p =0.07;  R2=0.04 | | |  |
| MVPA ( min∙week^-1^) | Education* | 0.64 ± 0.35 | | 0.16 |  | 0.59 ± 0.27 | | 0.15 |  |
|  | Age | 0.008 ± 0.08 | | 0.96 |  | 0.02 ± 0.06 | | 0.79 |  |
|  | Sex^ | 0.38 ± 0.16 | | 0.14 |  | 0.18 ± 0.12 | | 0.34 |  |
|  | PAR | 0.004 ± 0.08 | | 0.96 |  | 0.03 ± 0.06 | | 0.76 |  |
|  | Intercept | -0.72 ± 0.35 | | 0.15 |  | -0.56 ± 0.27 | | 0.15 |  |
|  | Model | F_6,149_=2.2; p =0.046;  R2=0.04 | | |  | F_6,149_=2.0; p =0.07;  R2=0.04 | | |  |
| TOTAL PA (MET:Min∙week-1) | Education* | 0.65 ± 0.35 | 0.15 | |  | 0.59 ± 0.29 | 0.15 | |  |
|  | Age | 0.01 ± 0.08 | 0.95 | |  | 0.01 ± 0.06 | 0.95 | |  |
|  | Sex^ | 0.37 ±0.16 | 0.15 | |  | 0.17 ± 0.12 | 0.40 | |  |
|  | PAR | -0.03 ± 0.08 | 0.83 | |  | 0.004 ± 0.06 | 0.95 | |  |
|  | Intercept | -0.72 ± 0.35 | 0.15 | |  | -0.56 ± 0.27 | 0.15 | |  |
|  | Model | F_6,149_=2.2; p =0.04;  R2=0.04 | | |  | F_6,149_=2.0; p =0.07;  R2=0.04 | | |  |
| Notes: Model: age, sex, education. ^#^ adjusted for multiple comparisons; ^ compared to men; high school certificate compared to university, ^+^ compared to those with diabetes . SDMT: Symbol Digit Modalities Test; DSBT: Digit-Span Backwards Task; PA: Physical activity; MVPA; moderate-to-vigorous physical activity. β are standardised. Table S1a | | | | | | | | | |

|  |  | Memory | |  |  | Fluid intelligence | |  |  |
| --- | --- | --- | --- | --- | --- | --- | --- | --- | --- |
|  | Variable | β ± SE | | p# |  | β ± SE | | p# |  |
| Light PA (min∙week^-1^) | Education* | -0.77 ± 1.31 | | 0.19 |  | 0.58 ± 0.27 | | 0.17 |  |
|  | Age | -0.14 ± 0.17 | | 0.86 |  | 0.02 ± 0.06 | | 0.80 |  |
|  | Sex^ | 0.05 ± 0.68 | | 0.16 |  | 0.16 ± 0.12 | | 0.42 |  |
|  | SWA | -0.11 ± 0.20 | | 0.67 |  | 0.02 ± 0.06 | | 0.80 |  |
|  | Intercept | -1.40 ± 0.01 | | 0.19 |  | -0.55 ± 0.28 | | 0.17 |  |
|  | Model | F_6,149_=2.2; p =0.04;  R^2^=0.05 | | |  | F_6,149_=2.0; p =0.07;  R^2^=0.04 | | |  |
| Moderate PA ( min∙week^-1^) | Education* | 0.64 ± 0.35 | | 0.16 |  | 0.58 ± 0.27 | | 0.14 |  |
|  | Age | 0.007 ± 0.08 | | 0.98 |  | 0.01 ± 0.06 | | 0.81 |  |
|  | Sex^ | 0.38 ± 0.17 | | 0.15 |  | 0.19 ± 0.13 | | 0.31 |  |
|  | SWA | 0.002 ± 0.08 | | 0.98 |  | 0.04 ± 0.06 | | 0.61 |  |
|  | Intercept | -0.72 ± 0.35 | | 0.15 |  | -0.56 ± 0.27 | | 0.14 |  |
|  | Model | F_6,149_=2.2; p =0.046;  R^2^=0.04 | | |  | F_6,149_=2.1; p =0.06;  R^2^=0.06 | | |  |
| Vigorous PA ( min∙week^-1^) | Education* | 0.63 ± 0.35 | | 0.17 |  | 0.58 ± 0.27 | | 0.13 |  |
|  | Age | 0.01 ± 0.08 | | 0.85 |  | 0.02 ± 0.06 | | 0.73 |  |
|  | Sex^ | 0.41 ± 0.16 | | 0.08 |  | 0.20 ± 0.12 | | 0.24 |  |
|  | SWA | 0.07 ± 0.08 | | 0.48 |  | 0.07 ± 0.06 | | 0.34 |  |
|  | Intercept | -0.74 ± 0.35 | | 0.13 |  | -0.57 ± 0.27 | | 0.13 |  |
|  | Model | F_6,149_=2.3; p =0.03;  R^2^=0.05 | | |  | F_6,149_=2.2; p =0.04;  R^2^=0.05 | | |  |
| MVPA ( min∙week^-1^) | Education* | 0.64 ± 0.35 | | 0.16 |  | 0.58 ± 0.27 | | 0.14 |  |
|  | Age | 0.01 ± 0.08 | | 0.92 |  | 0.02 ± 0.06 | | 0.79 |  |
|  | Sex^ | 0.39 ± 0.17 | | 0.15 |  | 0.21 ± 0.13 | | 0.27 |  |
|  | SWA | 0.02 ± 0.08 | | 0.92 |  | 0.05 ± 0.06 | | 0.48 |  |
|  | Intercept | -0.72 ± 0.35 | | 0.15 |  | -0.56 ± 0.27 | | 0.14 |  |
|  | Model | F_6,149_=2.2; p =0.05;  R^2^=0.04 | | |  | F_6,149_=2.1; p =0.06;  R^2^=0.04 | | |  |
| TOTAL PA (MET:mins∙week^-1^) | Education* | 0.62 ± 0.35 | 0.19 | |  | 0.56 ± 0.27 | 0.15 | |  |
|  | Age | 0.01 ± 0.08 | 0.88 | |  | 0.02 ± 0.06 | 0.73 | |  |
|  | Sex^ | 0.41 ± 0.16 | 0.10 | |  | 0.21 ± 0.13 | 0.23 | |  |
|  | SWA | 0.05 ± 0.08 | 0.62 | |  | 0.07 ±0.06 | 0.35 | |  |
|  | Intercept | -0.72 ± 0.35 | 0.15 | |  | -0.55 ± 0.27 | 0.15 | |  |
|  | Model | F_6,149_=2.2; p =0.04;  R^2^=0.05 | | |  | F_6,149_=2.2; p =0.04;  R^2^=0.05 | | |  |
| Notes: Model: age, sex, education. ^#^ adjusted for multiple comparisons; ^ compared to men; high school certificate compared to university, ^+^ compared to those with diabetes . SDMT: Symbol Digit Modalities Test; DSBT: Digit-Span Backwards Task; PA: Physical activity; MVPA; moderate-to-vigorous physical activity. β are standardised. Table S1b | | | | | | | | | |

Table S3b – The association between physical activity, measured by the SenseWear Armband^™^ , and memory and fluid intelligence

Table S4a – The association between physical activity, measured by the Physical Activity Recall survey, and cognitive function when controlling for age, sex, education, BMI, hypertension, diabetes, and audit class with interaction terms.

|  |  | Immediate recall |  |  | Delayed recall | |  |  | SDMT |  | |  | DSBT | |  |
| --- | --- | --- | --- | --- | --- | --- | --- | --- | --- | --- | --- | --- | --- | --- | --- |
|  |  | β ± SE | p# |  | β ± SE | | p# |  | β ± SE | p# | |  | β ± SE | | p# |
| Light PA (min∙week-1) | BMI | -0.12 ± 0.53 | 0.70 |  | -0.11 ± 0.10 | | 0.96 |  | -0.04 ± 0.10 | 0.86 | |  | 0.003 ± 0.09 | | 0.99 |
|  | Hypertension | -0.16 ± 0.53 | 0.93 |  | 0.22 ± 0.54 | | 0.96 |  | -0.38 ± 0.54 | 0.86 | |  | 0.81 ± 0.52 | | 0.85 |
|  | Audit class^Y^ | -0.80 ± 0.97 | 0.70 |  | 0.16 ± 0.99 | | 0.96 |  | 1.06 ± 1.00 | 0.86 | |  | -0.003 ± 0.09 | | 0.99 |
|  | Diabetes^+^ | -2.88 ± 3.81 | 0.70 |  | -0.46 ± 0.45 | | 0.96 |  | -0.95 ± 0.94 | 0.94 | |  | 3.94 ± 3.79 | | 0.87 |
|  | Education* | 0.96 ± 0.44 | 0.61 |  | 0.41 ± 0.45 | | 0.96 |  | 0.49 ± 0.45 | 0.86 | |  | 0.55 ± 0.44 | | 0.85 |
|  | Age | 0.10 ± 0.09 | 0.70 |  | -0.01 ± 0.09 | | 0.96 |  | -0.06 ± 0.09 | 0.86 | |  | 0.12 ± 0.09 | | 0.85 |
|  | Sex^ | 0.38 ± 0.19 | 0.61 |  | 0.46 ± 0.19 | | 0.49 |  | -0.09 ± 0.19 | 0.86 | |  | -0.02 ± 0.19 | | 0.99 |
|  | *PAR* | 8.72 ± 10.95 | 0.70 |  | 1.37 ± 11.16 | | 0.96 |  | 5.68 ± 11.32 | 0.86 | |  | -15.33 ± 10.89 | | 0.85 |
|  | Intercept | 2.06 ± 3.89 | 0.80 |  | -0.61.37 ± 3.97 | | 0.96 |  | 0.65 ± 4.02 | 0.94 | |  | -4.87 ± 3.87 | | 0.85 |
|  | *PAR* *BMI | 0.13 ± 0.12 | 0.70 |  | 0.07 ± 0.12 | | 0.96 |  | 0.12 ± 0.13 | 0.86 | |  |  | | 0.87 |
|  | *PAR* *Hypertension | -0.02 ± 1.32 | 0.99 |  | 0.82 ± 1.34 | | 0.96 |  | 0.53 ± 1.36 | 0.86 | |  | -0.10 ± 0.12 | | 0.87 |
|  | *PAR* *Audit Class | 2.07 ± 1.29 | 0.70 |  | 1.76 ± 1.32 | | 0.96 |  | -1.39 ± 1.33 | 0.86 | |  | 1.32 ± 1.31 | | 0.99 |
|  | *PAR* *Diabetes | -10.45 ± 10.86 | 0.70 |  | -3.56 ± 11.07 | | 0.96 |  | -4.20 ± 11.22 | 0.86 | |  | 14.23 ± 10.80 | | 0.99 |
|  | *PAR* *Education | 0.72 ± 0.83 | 0.70 |  | -0.004 ± 0.85 | | 0.99 |  | 0.01 ± 0.86 | 0.99 | |  | 0.008 ± 0.83 | | 0.99 |
|  | *PAR* *Age | 0.15 ± 0.014 | 0.70 |  | 0.10 ± 0.29 | | 0.96 |  | 0.21 ± 0.15 | 0.86 | |  | -0.18 ± 0.14 | | 0.85 |
|  | *PAR* *Sex | -0.04 ± 0.29 | 0.95 |  | 0.03 ± 0.29 | | 0.96 |  | -0.31 ± 0.30 | 0.86 | |  | 0.22 ± 0.29 | | 0.87 |
|  | Model | F_27,128_=1.1; p =0.35;  R2=0.02, ∆ R^2^ = | |  | F_27,128_=0.9; p =0.64;  R2=-0.02, ∆ R^2^ = | | |  | F_27,128_=0.7; p =0.83;  R2=-0.05, ∆ R^2^ = | | |  | F_27,128_=1.2; p =0.28;  R2=0.03, ∆ R^2^ = | | |
| Moderate PA  ( min∙week-1) | BMI | -0.11 ± 0.09 | 0.40 |  | -0.11 ± 0.09 | | 0.53 |  | 0.01 ± 0.10 | 0.97 | |  | 0.02 ± 0.10 | | 0.88 |
|  | Hypertension | -0.24 ± 0.41 | 0.66 |  | -0.02 ± 0.42 | | 0.49 |  | -0.53 ± 0.43 | 0.97 | |  | 0.42 ± 0.43 | | 0.68 |
|  | Audit class^Y^ | -0.40 ± 0.58 | 0.71 |  | 0.52 ± 0.92 | | 0.95 |  | 0.68 ± 0.96 | 0.97 | |  | 0.57 ± 0.94 | | 0.68 |
|  | Diabetes^+^ | 3.18 ± 2.89 | 0.43 |  | 1.56 ± 2.93 | | 0.76 |  | 1.08 ± 3.06 | 0.97 | |  | -4.06 ± 3.00 | | 0.68 |
|  | Education* | 0.91 ± 0.41 | 0.40 |  | 0.48 ± 0.41 | | 0.53 |  | 0.50 ± 0.43 | 0.97 | |  | 0.64 ± 0.42 | | 0.68 |
|  | Age | 0.08 ± 0.09 | 0.52 |  | -0.02 ± 0.09 | | 0.90 |  | -0.08 ± 0.09 | 0.97 | |  | 0.09 ± 0.09 | | 0.68 |
|  | Sex^ | 0.39 ± 0.18 | 0.40 |  | 0.45 ± 0.19 | | 0.44 |  | -0.12 ± 0.19 | 0.97 | |  | 0.01 ± 0.19 | | 0.96 |
|  | *PAR* | -9.07 ± 7.14 | 0.40 |  | -5.53 ± 7.24 | | 0.69 |  | -0.95 ± 7.57 | 0.97 | |  | 8.43 ± 7.41 | | 0.68 |
|  | Intercept | -4.18 ± 2.99 | 0.40 |  | -2.90 ± 3.04 | | 0.56 |  | -1.05 ± 3.17 | 0.97 | |  | 3.58 ± 3.11 | | 0.68 |
|  | *PAR* *BMI | 0.07 ± 0.11 | 0.64 |  | 0.02 ± 0.12 | | 0.90 |  | 0.08 ± 0.12 | 0.97 | |  | -0.02 ± 0.12 | | 0.88 |
|  | *PAR* *Hypertension | -0.52 ± 0.46 | 0.42 |  | -0.26 ± 0.46 | | 0.76 |  | 0.53 ± 0.48 | 0.97 | |  | -0.55 ± 0.47 | | 0.68 |
|  | *PAR* *Audit Class | 3.76 ± 2.23 | 0.40 |  | 3.20 ± 2.26 | | 0.49 |  | -1.43 ± 2.36 | 0.97 | |  | 0.59 ± 2.31 | | 0.88 |
|  | *PAR* *Diabetes | 6.53 ± 6.94 | 0.51 |  | 2.46 ± 7.04 | | 0.85 |  | 2.02 ± 7.35 | 0.97 | |  | -7.63 ± 7.20 | | 0.68 |
|  | *PAR* *Education | 0.95 ± 0.66 | 0.40 |  | 0.66 ± 0.67 | | 0.56 |  | 0.13 ± 0.70 | 0.97 | |  | 0.52 ± 0.69 | | 0.68 |
|  | *PAR* *Age | -0.04 ± 0.09 | 0.71 |  | 0.02 ± 0.42 | | 0.88 |  | 0.12 ± 0.09 | 0.97 | |  | 0.02 ± 0.09 | | 0.88 |
|  | *PAR* *Sex | 0.35 ± 0.22 | 0.40 |  | 0.36 ± 0.22 | | 0.49 |  | 0.01 ± 0.23 | 0.97 | |  | 0.28 ± 0.23 | | 0.68 |
|  | Model | F_27,128_=1.3; p =0.17;  R2=0.05, ∆ R^2^ = | |  | F_27,128_=1.3; p =0.17;  R2=0.05, ∆ R^2^ = | | |  | F_27,128_=0.6; p =0.92;  R2=-0.07, ∆ R^2^ = | | |  | F_27,128_=0.9; p =0.66;  R2=-0.02, ∆ R^2^ = | | |
| Vigorous PA  ( min∙week-1) | BMI | -0.11 ± 0.09 | 0.56 |  | -0.10 ± 0.10 | | 0.88 |  | 0.02 ± 0.10 | 0.90 | |  | 0.05 ± 0.10 | | 0.83 |
|  | Hypertension | -0.33 ± 0.54 | 0.62 |  | 0.05 ± 0.54 | | 0.99 |  | -0.42 ± 0.57 | 0.90 | |  | 0.23 ± 0.55 | | 0.83 |
|  | Audit class^Y^ | -11.77 ± 7.02 | 0.56 |  | -10.23 ± 7.09 | | 0.88 |  | -2.34 ± 7.44 | 0.90 | |  | -12.96 ± 7.21 | | 0.83 |
|  | Diabetes^+^ | 0.67 ± 0.73 | 0.56 |  | 0.80 ± 0.74 | | 0.88 |  | 0.34 ± 0.77 | 0.90 | |  | -0.85 ± 0.75 | | 0.83 |
|  | Education* | 0.97 ± 0.42 | 0.30 |  | 0.45 ± 0.42 | | 0.88 |  | 0.61 ± 0.44 | 0.90 | |  | 0.71 ± 0.43 | | 0.83 |
|  | Age | 0.07 ± 0.09 | 0.56 |  | -0.03 ± 0.09 | | 0.95 |  | -0.07 ± 0.09 | 0.90 | |  | 0.10 ± 0.09 | | 0.83 |
|  | Sex^ | 0.49 ± 0.19 | 0.30 |  | 0.54 ± 0.19 | | 0.15 |  | -0.05 ± 0.20 | 0.90 | |  | 0.05 ± 0.20 | | 0.83 |
|  | *PAR* | -0.44 ± 2.91 | 0.88 |  | -0.40 ± 2.94 | | 0.99 |  | -2.43 ± 3.08 | 0.90 | |  | 3.60 ± 2.99 | | 0.83 |
|  | Intercept | -0.59 ± 1.30 | 0.72 |  | -1.14 ± 1.31 | | 0.88 |  | -1.58 ± 1.37 | 0.90 | |  | 0.62 ± 1.33 | | 0.83 |
|  | *PAR* *BMI | 0.03 ± 0.17 | 0.88 |  | -0.002 ± 0.17 | | 0.99 |  | 0.03 ± 0.18 | 0.90 | |  | -0.04 ± 0.17 | | 0.04 |
|  | *PAR* *Hypertension | -0.50 ± 1.15 | 0.72 |  | 0.02 ± 1.16 | | 0.99 |  | 0.66 ± 1.22 | 0.90 | |  | -1.07 ± 1.18 | | 0.83 |
|  | *PAR* *Audit Class | 6.62 ± 5.85 | 0.56 |  | 6.34 ± 5.91 | | 0.88 |  | 0.49 ± 6.20 | 0.90 | |  | 9.44 ± 6.01 | | 0.83 |
|  | *PAR* *Diabetes | 1.80 ± 0.34 | 0.56 |  | 1.08 ± 1.35 | | 0.88 |  | 0.26 ± 1.41 | 0.90 | |  | -1.40 ± 1.37 | | 0.83 |
|  | *PAR* *Education | 0.69 ± 0.66 | 0.56 |  | 0.28 ± 0.66 | | 0.95 |  | -0.28 ± 0.69 | 0.90 | |  | -0.02 ± 0.67 | | 0.98 |
|  | *PAR* *Age | 0.13 ± 0.14 | 0.56 |  | 0.09 ± 0.14 | | 0.92 |  | 0.10 ± 0.15 | 0.90 | |  | 0.18 ± 0.15 | | 0.83 |
|  | *PAR* *Sex | 0.58 ± 0.33 | 0.56 |  | 0.62 ± 0.33 | | 0.76 |  | -0.07 ± 0.35 | 0.90 | |  | 0.09 ± 0.34 | | 0.84 |
|  | Model | F_27,128_=1.2; p =0.23;  R2=0.04, ∆ R^2^ = | |  | F_27,128_=1.1; p =0.33;  R2=0.02, ∆ R^2^ = | | |  | F_27,128_=0.6; p =0.95;  R2=-0.08, ∆ R^2^ = | | |  | F_27,128_=0.9; p =0.59  R2=-0.01, ∆ R^2^ = | | |
| MVPA  ( min∙week-1) | BMI | -0.12 ± 0.09 | 0.45 |  | -0.12 ± 0.09 | | 0.46 |  | 0.02 ± 0.10 | 0.98 | |  | 0.04 ± 0.10 | | 0.81 |
|  | Hypertension | -0.33 ± 0.43 | 0.63 |  | -0.03 ± 0.43 | | 0.97 |  | -0.52 ± 0.46 | 0.98 | |  | 0.35 ± 0.45 | | 0.55 |
|  | Audit class^Y^ | -2.27 ± 2.14 | 0.54 |  | -0.63 ± 2.14 | | 0.86 |  | -0.11 ± 2.28 | 0.98 | |  | -2.48 ± 2.22 | | 0.55 |
|  | Diabetes^+^ | 0.98 ± 0.77 | 0.45 |  | 0.87 ± 0.77 | | 0.46 |  | 0.44 ± 0.82 | 0.98 | |  | -1.27 ± 0.80 | | 0.55 |
|  | Education* | 0.93 ± 0.39 | 0.29 |  | 0.45 ± 0.39 | | 0.46 |  | 0.48 ± 0.42 | 0.98 | |  | 0.66 ± 0.41 | | 0.55 |
|  | Age | 0.06 ± 0.09 | 0.63 |  | -0.03 ± 0.09 | | 0.86 |  | -0.05 ± 0.09 | 0.98 | |  | 0.08 ± 0.09 | | 0.55 |
|  | Sex^ | 0.43 ± 0.18 | 0.29 |  | 0.50 ± 0.18 | | 0.18 |  | -0.02 ± 0.19 | 0.98 | |  | 0.04 ± 0.19 | | 0.94 |
|  | *PAR* | -4.15 ± 2.83 | 0.45 |  | -4.65 ± 2.83 | | 0.46 |  | 0.20 ± 3.01 | 0.98 | |  | 4.40 ± 2.94 | | 0.55 |
|  | Intercept | -2.07 ± 1.32 | 0.45 |  | -2.64 ± 1.32 | | 0.39 |  | -0.42 ± 1.41 | 0.98 | |  | 1.49 ± 1.7 | | 0.55 |
|  | *PAR* *BMI | 0.04 ± 0.12 | 0.77 |  | -0.001 ± 0.12 | | 0.99 |  | 0.06 ± 0.12 | 0.98 | |  | -0.05 ±0.12 | | 0.81 |
|  | *PAR* *Hypertension | -0.44 ± 0.56 | 0.63 |  | -0.12 ± 0.56 | | 0.89 |  | 0.65 ± 0.59 | 0.98 | |  | -0.60 ± 0.58 | | 0.55 |
|  | *PAR* *Audit Class | 4.16 ± 3.03 | 0.45 |  | 4.78 ± 3.03 | | 0.46 |  | 0.07 ± 3.22 | 0.98 | |  | 0.43 ± 3.15 | | 0.95 |
|  | *PAR* *Diabetes | 2.03 ± 1.59 | 0.45 |  | 1.22 1.59 | | 0.59 |  | 0.52 ± 1.69 | 0.98 | |  | -1.62 ± 1.65 | | 0.55 |
|  | *PAR* *Education | 0.74 ± 0.53 | 0.45 |  | 0.46 ± 0.57 | | 0.54 |  | -0.22 ± 0.57 | 0.98 | |  | 0.06 ± 0.55 | | 0.95 |
|  | *PAR* *Age | 0.05 ± 0.10 | 0.71 |  | 0.10 ± 0.10 | | 0.51 |  | 0.14 ± 0.11 | 0.98 | |  | 0.12 ± 0.11 | | 0.55 |
|  | *PAR* *Sex | 0.54 ± 0.25 | 0.29 |  | 0.61 ± 0.25 | | 0.21 |  | 0.08 ± 0.26 | 0.98 | |  | 0.24 ± 0.26 | | 0.55 |
|  | Model | F_27,128_=1.4; p =0.13;  R2=0.06, ∆ R^2^ = | |  | F_27,128_=1.4; p =0.13;  R2=0.06, ∆ R^2^ = | | |  | F_27,128_=0.7; p =0.90;  R2=-0.06, ∆ R^2^ = | | |  | F_27,128_=0.9 p =0.58;  R2=-0.01, ∆ R^2^ = | | |
| MET_mins | BMI | -0.12 ± 0.09 | 0.43 |  | -0.11 ± 0.09 | 0.39 | |  | 0.003 ± 0.10 | | 0.98 |  | 0.05 ± 0.10 | 0.71 | |
|  | Hypertension | -0.44 ± 0.49 | 0.43 |  | -0.06 ± 0.49 | 0.93 | |  | -0.43 ±0.52 | | 0.98 |  | 0.29 ± 0.50 | 0.71 | |
|  | Audit class^Y^ | -1.22 ± 1.64 | 0.49 |  | 0.26 ± 1.64 | 0.93 | |  | -0.37 ± 1.74 | | 0.98 |  | -1.45 ± 1.69 | 0.65 | |
|  | Diabetes^+^ | 1.04 ± 0.96 | 0.43 |  | 0.89 ± 0.81 | 0.41 | |  | 0.44 ± 0.86 | | 0.98 |  | -1.36 ± 0.84 | 0.65 | |
|  | Education* | 0.97 ± 0.40 | 9.28 |  | 0.47 ± 0.40 | 0.39 | |  | 0.47 ± 0.43 | | 0.98 |  | 0.66 ± 0.41 | 0.65 | |
|  | Age | 0.10 ± 0.09 | 0.43 |  | -0.004 ±0.09 | 0.96 | |  | -0.05 ± 0.19 | | 0.98 |  | 0.09 ±0.09 | 0.65 | |
|  | Sex^ | 0.43 ± 0.18 | 0.28 |  | 0.50 ± 0.18 | 0.19 | |  | -0.06 ± 0.19 | | 0.98 |  | 0.03 ± 0.19 | 0.89 | |
|  | *PAR* | -5.47 ± 3.36 | 0.43 |  | -5.89 ±3.36 | *0.27* | |  | *2.48* ± 3.56 | | 0.98 |  | *5.36* ± 3.46 | *0.65* | |
|  | Intercept | -2.52 ± 1.41 | 0.43 |  | -3.08 ± 1.41 | 0.27 | |  | 0.15 ± 1.50 | | 0.98 |  | 1.67 ± 1.45 | 0.65 | |
|  | *PAR* *BMI | 0.04 ± 0.12 | 0.70 |  | -0.03 ± 0.12 | 0.92 | |  | 0.04 ± 0.12 | | 0.98 |  | -0.06 ± 0.12 | 0.71 | |
|  | *PAR* *Hypertension | -0.65 ± 0.80 | 0.47 |  | -0.20 ± 0.80 | 0.92 | |  | 0.67 ± 0.12 | | 0.98 |  | -0.64 ± 0.83 | 0.67 | |
|  | *PAR* *Audit Class | 4.67 ± 2.75 | 0.43 |  | 5.56 ± 2.75 | 0.27 | |  | -2.40 ± 2.92 | | 0.98 |  | -1.44 ± 2.83 | 0.71 | |
|  | *PAR* *Diabetes | 2.16 ± 1.88 | 0.43 |  | 1.16 ± 1.88 | 0.74 | |  | 0.39 ± 1.99 | | 0.98 |  | -2.04 ± 1.93 | 0.65 | |
|  | *PAR* *Education | 0.78 ± 0.59 | 0.43 |  | 0.35 ± 0.59 | 0.74 | |  | -0.30 ± 0.62 | | 0.98 |  | -0.08 ± 0.60 | 0.89 | |
|  | *PAR* *Age | 0.12 ± 0.12 | 0.43 |  | 0.14 ± 0.12 | 0.39 | |  | 0.16 ± 0.13 | | 0.98 |  | 0.06 ± 0.13 | 0.71 | |
|  | *PAR* *Sex | 0.41 ± 0.26 | 0.43 |  | 0.51 ± 0.26 | 0.27 | |  | 0.11 ± 0.28 | | 0.98 |  | 0.24 ± 0.27 | 0.65 | |
|  | Model | F_27,128_=1.3; p =0.18;  R2=0.05, ∆ R^2^ = 0.05 | |  | F_27,128_=1.3; p =0.18;  R2=0.05, ∆ R^2^ = 0.01 | | |  | F_27,128_=0.6; p =0.93;  R2=-0.07, ∆ R^2^ = 0.072 | | |  | F_27,128_=0.9; p =0.55;  R2=-0.01, ∆ R^2^ = -0.013 | | |
| Notes: Model: age, sex, education, BMI, hypertension, diabetes, and audit class, with interactions. ^#^ adjusted for multiple comparisons; ^ compared to men; high school certificate compared to university; ^+^ compared to those with diabetes; ^Y^heavy drinkers compared to abstainers. SDMT: Symbol Digit Modalities Test; DSBT: Digit-Span Backwards Task; PA: Physical activity; MVPA; moderate-to-vigorous physical activity. β are standardised. ∆ R^2^ compared to model presented in Table S1a | | | | | | | | | | | | | | | |

Table S4b – The association between physical activity, measured by the SenseWear Armband™, and cognitive function when controlling for age, sex, education, BMI, hypertension, diabetes, and audit class with interaction terms.

|  |  | Immediate recall |  |  | Delayed recall |  | |  | SDMT |  |  | DSBT | |  |
| --- | --- | --- | --- | --- | --- | --- | --- | --- | --- | --- | --- | --- | --- | --- |
|  |  | β ± SE | p# |  | β ± SE | p# | |  | β ± SE | p# |  | β ± SE | | p# |
| Light PA (min∙week-1) | BMI | -0.08 ± 0.11 | 0.86 |  | -0.07 ± 0.11 | 0.99 | |  | -0.09 ± 0.11 | 0.79 |  | 0.07 ± 0.11 | | 0.90 |
|  | Hypertension | -0.17 ± 0.49 | 0.93 |  | -0.02 ± 0.50 | 0.99 | |  | -0.26 ± 0.49 | 0.84 |  | 0.70 ± 0.48 | | 0.90 |
|  | Audit class^Y^ | -0.88 ± 0.92 | 0.86 |  | 0.09 ± 0.94 | 0.99 | |  | 1.56 ± 0.93 | 0.40 |  | 0.89 ± 0.92 | | 0.91 |
|  | Diabetes^+^ | 0.92 ± 0.80 | 0.86 |  | 0.83 ± 0.82 | 0.99 | |  | 0.51 ± 0.80 | 0.82 |  | -1.29 ± 0.80 | | 0.90 |
|  | Education* | 0.80 ± 0.42 | 0.86 |  | 0.29 ± 0.43 | 0.99 | |  | 0.04 ± 0.47 | 0.71 |  | 0.48 ± 0.42 | | 0.90 |
|  | Age | 0.06 ± 0.09 | 0.86 |  | -0.02 ± 0.09 | 0.99 | |  | -0.07 ± 0.09 | 0.79 |  | 0.11 ± 0.09 | | 0.90 |
|  | Sex^ | 0.38 ± 0.19 | 0.86 |  | 0.45 ± 0.19 | 0.59 | |  | -0.14 ± 0.19 | 0.79 |  | -0.08 ± 0.19 | | 0.90 |
|  | SWA | -1.34 ± 1.66 | 0.86 |  | -0.39 ± 1.69 | 0.99 | |  | 0.10 ± 1.66 | 0.95 |  | 1.06 ± 1.65 | | 0.90 |
|  | Intercept | -1.44 ± 1.15 | 0.86 |  | -1.48 ± 1.17 | 0.99 | |  | -1.37 ± 1.12 | 0.66 |  | 0.32 ± 1.14 | | 0.90 |
|  | SWA*BMI | 0.15 ± 0.10 | 0.86 |  | 0.09 ± 0.10 | 0.99 | |  | -0.06 ± 0.10 | 0.82 |  | 0.13 ± 0.10 | | 0.90 |
|  | SWA*Hypertension | 0.05 ± 0.77 | 0.95 |  | -0.07 ± 0.79 | 0.99 | |  | 0.42 ± 0.78 | 0.84 |  | 0.19 ± 0.77 | | 0.90 |
|  | SWA*Audit Class | 1.03 ± 1.09 | 0.86 |  | 0.58 ± 1.11 | 0.99 | |  | 0.20 ± 1.10 | 0.95 |  | 1.48 ± 1.08 | | 0.90 |
|  | SWA*Diabetes | 1.01 ± 1.05 | 0.86 |  | 0.35 ± 1.07 | 0.99 | |  | -0.11 ± 1.04 | 0.95 |  | -1.43 ± 1.04 | | 0.90 |
|  | SWA*Education | 0.41 ± 0.41 | 0.86 |  | 0.17 ± 0.41 | 0.99 | |  | -0.36 ± 0.40 | 0.79 |  | 0.14 ± 0.40 | | 0.90 |
|  | SWA*Age | -0.07 ± 0.09 | 0.86 |  | -0.001 ± 0.09 | 0.99 | |  | 0.17 ± 0.09 | 0.40 |  | -0.05 ± 0.09 | | 0.90 |
|  | SWA*Sex | -0.02 ± 0.21 | 0.95 |  | 0.02 ± 0.22 | 0.99 | |  | -0.46 ± 0.21 | 0.40 |  | -0.17 ± 0.21 | | 0.90 |
|  | Model | F_27,128_=1.1; p =0.39;  R2=0.01, ∆ R^2^ = -0.04 | |  | F_27,128_=0.9; p =0.6;  R2=-0.02, ∆ R^2^ = -0.06 | | |  | F_27,128_=1.0; p =0.41;  R2=-0.01, ∆ R^2^ = 0.097 | |  | F_27,128_=1.2; p =0.28;  R2=-0.03, ∆ R^2^ = 0.0297 | | |
| Moderate PA  ( min∙week-1) | BMI | -0.15 ± 0.13 | 0.66 |  | -0.15 ± 0.14 | 0.94 | |  | -0.03 ± 0.14 | 0.94 |  | -0.05 ± 0.13 | | 0.98 |
|  | Hypertension | -0.06 ± 0.42 | 0.94 |  | 0.14 ± 0.42 | 0.94 | |  | -0.49 ± 0.43 | 0.76 |  | 0.76 ± 0.42 | | 0.75 |
|  | Audit class^Y^ | -10.28 ± 10.91 | 0.66 |  | -6.07 ± 11.11 | 0.94 | |  | -4.32 ± 11.18 | 0.89 |  | -14.36 ± 10.85 | | 0.75 |
|  | Diabetes^+^ | 0.88 ± 0.78 | 0.66 |  | 0.80 ± 0.80 | 0.94 | |  | 0.43 ± 0.80 | 0.87 |  | -1.12 ± 0.78 | | 0.75 |
|  | Education* | 1.05 ± 0.43 | 0.48 |  | 0.54 ± 0. 44 | 0.94 | |  | 0.54 ± 0.44 | 0.76 |  | 0.75 ± 0.43 | | 0.75 |
|  | Age | 0.07 ± 0.09 | 0.66 |  | -0.02 ± 0.09 | 0.94 | |  | -0.04 ± 0.09 | 0.89 |  | 0.10 ± 0.09 | | 0.85 |
|  | Sex^ | 037 ± 0.21 | 0.66 |  | 0.40 ± 0.22 | 0.94 | |  | -0.15 ± 0.22 | 0.81 |  | -0.06 ± 0.21 | | 0.98 |
|  | SWA | 0.08 ± 1.66 | 0.96 |  | 0.17 ± 1.69 | 0.94 | |  | -0.09 ± 1.70 | 0.81 |  | 1.20 ± 1.65 | | 0.98 |
|  | Intercept | -1.32 ± 1.26 | 0.66 |  | -1.43 ± 1.28 | 0.94 | |  | -1.44 ± 1.29 | 0.76 |  | -0.10 ± 1.25 | | 0.98 |
|  | SWA*BMI | 0.02 ± 0.13 | 0.94 |  | 0.05 ± 0.13 | 0.94 | |  | -0.01 ± 0.14 | 0.98 |  | -0.03 ± 0.13 | | 0.98 |
|  | SWA*Hypertension | -0.06 ± 0.47 | 0.94 |  | 0.31 ± 0.48 | 0.94 | |  | 0.20 ± 0.48 | 0.89 |  | 0.15 ± 0.47 | | 0.98 |
|  | SWA*Audit Class | 3.32 ± 5.39 | 0.66 |  | 1.97 ± 5.49 | 0.94 | |  | 4.24 ± 5.52 | 0.78 |  | 7.14 ± 5.36 | | 0.75 |
|  | SWA*Diabetes | 0.53 ± 0.79 | 0.66 |  | 0.13 ± 0.81 | 0.94 | |  | 0.05 ± 0.53 | 0.98 |  | -1.12 ± 0.79 | | 0.75 |
|  | SWA*Education | 0.57 ± 0.52 | 0.66 |  | 0.22 ± 0.53 | 0.94 | |  | 0.02 ± 0.53 | 0.98 |  | 0.11 ± 0.51 | | 0.98 |
|  | SWA*Age | -0.04 ± 0.09 | 0.78 |  | -0.04 ± 0.56 | 0.94 | |  | 0.11 ± 0.10 | 0.76 |  | -0.03 ± 0.09 | | 0.98 |
|  | SWA*Sex | -0.28 ± 0.24 | 0.66 |  | -0.19 ± 0.24 | 0.94 | |  | -0.23 ± 0.24 | 0.76 |  | -0.50 ± 0.24 | | 0.75 |
|  | Model | F27,128=1.0; p =0.4;  R2=0.01, ∆ R^2^ = -0.04 | |  | F27,128=0.8; p =0.74;  R2=-0.04, ∆ R^2^ = -0.012 | | |  | F27,128=0.7; p =0.82;  R2=-0.05, ∆ R^2^ = 0.054 | |  | F27,128=1.1; p =0.38;  R2=0.01, ∆ R^2^ = 0.007 | | |
| Vigorous PA  ( min∙week-1) | BMI | -0.11 ± 0.14 | 0.75 |  | -0.10 ± 0.14 | 0.86 | |  | 0.11 ± 0.14 | 0.99 |  | 0.09 ± 0.14 | | 0.81 |
|  | Hypertension | -0.15 ± 0.43 | 0.88 |  | -0.06 ± 0.43 | 0.96 | |  | -0.54 ± 0.45 | 0.99 |  | 0.48 ± 0.43 | | 0.81 |
|  | Audit class^Y^ | -0.93 ± 0.92 | 0.74 |  | -0.41 ± 0.57 | 0.93 | |  | 1.03 ± 0.95 | 0.99 |  | 0.69 ± 0.91 | | 0.81 |
|  | Diabetes^+^ | 1.25 ± 1.03 | 0.74 |  | 0.84 ± 1.04 | 0.86 | |  | 0.39 ± 1.07 | 0.99 |  | -1.80 ± 1.02 | | 0.81 |
|  | Education* | 0.98 ± 0.41 | 0.49 |  | 0.56 ± 0.41 | 0.86 | |  | 0.38 ± 0.42 | 0.99 |  | 0.71 ± 0.40 | | 0.81 |
|  | Age | 0.08 ± 0.09 | 0.75 |  | -0.001 ± 0.09 | 0.99 | |  | -0.05 ± 0.09 | 0.99 |  | 0.11 ± 0.09 | | 0.81 |
|  | Sex^ | 0.40 ± 0.21 | 0.74 |  | -0.82 ± 2.74 | 0.86 | |  | -0.001 ± 0.21 | 0.99 |  | 0.11 ± 0.21 | | 0.81 |
|  | SWA | -2.47 ± 2.71 | 0.75 |  | -0.82 ± 2.74 | 0.96 | |  | 0.42 ± 2.81 | 0.99 |  | 1.86 ± 2.69 | | 0.81 |
|  | Intercept | -1.93 ± 1.32 | 0.74 |  | -1.41 ± 1.34 | 0.86 | |  | -0.61 ± 1.37 | 0.99 |  | 0.90 ± 1.31 | | 0.81 |
|  | SWA*BMI | 0.02 ± 0.19 | 0.91 |  | 0.10 ± 0.20 | 0.91 | |  | 0.21 ± 0.20 | 0.99 |  | 0.11 ± 0.19 | | 0.81 |
|  | SWA*Hypertension | 0.18 ± 0.50 | 0.88 |  | 0.59 ± 0.51 | 0.86 | |  | -0.60 ± 0.52 | 0.99 |  | 0.42 ± 0.50 | | 0.81 |
|  | SWA*Audit Class | -1.91 ± 1.69 | 0.74 |  | -1.57 ± 1.71 | 0.86 | |  | 0.24 ± 1.75 | 0.99 |  | -1.52 ± 1.68 | | 0.81 |
|  | SWA*Diabetes | 2.02 ± 2.42 | 0.75 |  | 0.39 ± 2.44 | 0.96 | |  | 0.03 ± 2.50 | 0.99 |  | -2.71 ± 2.40 | | 0.81 |
|  | SWA*Education | 0.91 ± 0.65 | 0.74 |  | 0.83 ± 0.66 | 0.86 | |  | 0.03 ± 0.67 | 0.99 |  | 0.28 ± 0.64 | | 0.83 |
|  | SWA*Age | -0.02 ± 0.09 | 0.91 |  | -0.05 ± 0.09 | 0.91 | |  | 0.003 ± 0.09 | 0.99 |  | -0.12 ± 0.08 | | 0.81 |
|  | SWA*Sex | -0.16 ± 0.22 | 0.78 |  | -0.22 ± 0.22 | 0.86 | |  | -0.05 ± 0.23 | 0.99 |  | 0.03 ± 0.22 | | 0.91 |
|  | Model | F27,128=1.0; p =0.4;  R^2^=<-0.001, ∆ R^2^ =>0.061 | |  | F27,128=0.9; p =0.63;  R2=-0.02, ∆ R^2^ = 0.05 | | |  | F27,128=0.6; p =0.93;  R2=-0.07, ∆ R^2^ = 0.075 | |  | F27,128=1.1; p =0.36;  R2=0.02, ∆ R^2^ = < 0.019 | | |
| MVPA  ( min∙week-1) | BMI | -0.15 ± 0.14 | 0.60 |  | -0.14 ± 0.14 | 0.91 | |  | -0.01 ± 0.14 | 0.98 |  | -0.03 ± 0.14 | | 0.97 |
|  | Hypertension | -0.05 ± 0.47 | 0.97 |  | 0.14 ± 0.42 | 0.91 | |  | -0.52 ± 0.43 | 0.74 |  | 0.77 ± 0.42 | | 0.82 |
|  | Audit class^Y^ | -26.63 ± 30.79 | 0.60 |  | -17.22 ± 31.34 | 0.91 | |  | -14.97 ± 31.55 | 0.74 |  | -38.55 ± 30.62 | | 0.82 |
|  | Diabetes^+^ | 0.92 ± 0.79 | 0.60 |  | 0.82 ± 0.80 | 0.91 | |  | 0.43 ± 0.81 | 0.90 |  | -1.15 ± 0.78 | | 0.82 |
|  | Education* | 1.06 ± 0.43 | 0.42 |  | 0.56 ± 0.44 | 0.91 | |  | 0.53 ± 0.44 | 0.74 |  | 0.73 ± 0.43 | | 0.82 |
|  | Age | 0.08 ± 0.09 | 0.60 |  | -0.02 ± 0.09 | 0.91 | |  | -0.03 ± 0.09 | 0.98 |  | 0.10 ± 0.09 | | 0.82 |
|  | Sex^ | 0.38 ± 0.21 | 0.60 |  | 0.41 ± 0.22 | 0.91 | |  | -0.12 ± 0.22 | 0.90 |  | -0.02 ± 0.21 | | 0.97 |
|  | SWA | 0.04 ± 1.52 | 0.98 |  | 0.25 ± 1.55 | 0.91 | |  | -0.72 ± 1.56 | 0.90 |  | 1.14 ± 1.51 | | 0.97 |
|  | Intercept | -1.38 ± 1.22 | 0.60 |  | -1.43 ± 1.25 | 0.91 | |  | -1.29 ± 0.26 | 0.74 |  | -0.04 ± 1.22 | | 0.97 |
|  | SWA*BMI | 0.02 ± 0.13 | 0.97 |  | 0.06 ± 0.13 | 0.91 | |  | 0.004 ± 0.13 | 0.98 |  | -0.02 ± 0.13 | | 0.97 |
|  | SWA*Hypertension | -0.03 ± 0.44 | 0.98 |  | 0.33 ± 0.45 | 0.91 | |  | 0.08 ± 0.45 | 0.98 |  | 0.21 ± 0.44 | | 0.97 |
|  | SWA*Audit Class | 12.30 ± 16.23 | 0.60 |  | 7.96 ± 16.52 | 0.91 | |  | 9.84 ± 16.63 | 0.90 |  | 20.42 ± 16.14 | | 0.82 |
|  | SWA*Diabetes | 0.53 ± 0.80 | 0.62 |  | 0.09 ± 0.82 | 0.91 | |  | 0.02 ± 0.82 | 0.98 |  | -1.10 ± 0.80 | | 0.82 |
|  | SWA*Education | 0.60 ± 0.50 | 0.60 |  | 0.29 ± 0.51 | 0.91 | |  | 0.04 ± 0.51 | 0.98 |  | 0.10 ± 0.50 | | 0.97 |
|  | SWA*Age | -0.04 ± 0.09 | 0.71 |  | -0.05 ± 0.09 | 0.91 | |  | 0.11 ± 0.09 | 0.74 |  | -0.06 ± 0.09 | | 0.97 |
|  | SWA*Sex | -0.28 ± 0.23 | 0.60 |  | -0.22 ± 0.23 | 0.91 | |  | -0.24 ± 0.23 | 0.74 |  | -0.42 ± 0.23 | | 0.82 |
|  | Model | F27,128=1.0; p =0.4;  R2=0.005, ∆ R^2^ = 0.045 | |  | F27,128=0.9; p =0.71;  R2=-0.03, ∆ R^2^ = 0.08 | | |  | F27,128=0.7; p =0.80;  R2=-0.05, ∆ R^2^ =-0.06 | |  | F27,128=1.1; p =0.36;  R2=0.01, ∆ R^2^ = 0.06 | | |
| TOTAL PA (MET:mins∙week^-1^) | BMI | -0.06 ± 0.14 | 0.75 |  | -0.07 ±0.14 | | 0.98 |  | 0.04 ± 0.14 | 0.95 |  | 0.12 ± 0.13 | 0.72 | |
|  | Hypertension | -0.12 ± 0.41 | 0.83 |  | 0.11 ± 0.42 | | 0.98 |  | -0.48 ± 0.42 | 0.64 |  | 0.68 ± 0.40 | 0.66 | |
|  | Audit class^Y^ | -5.23 ± 0.70 | 0.70 |  | -3.27 ± 4.47 | | 0.98 |  | -1.27 ± 4.43 | 0.95 |  | -4.84 ± 4.29 | 0.68 | |
|  | Diabetes^+^ | 0.85 ± 0.78 | 0.70 |  | 0.82 ± 0.80 | | 0.98 |  | 0.39 ± 0.79 | 0.92 |  | -1.13 ± 0.76 | 0.66 | |
|  | Education* | 1.00 ± 0.43 | 0.59 |  | 0.54 ± 0.44 | | 0.98 |  | 0.50 ± 0.43 | 0.64 |  | 0.65 ± 0.42 | 0.66 | |
|  | Age | 0.09 ± 0.09 | 0.70 |  | -0.004 ± 0.09 | | 0.98 |  | -0.22 ± 0.09 | 0.95 |  | 0.14 ± 0.09 | 0.66 | |
|  | Sex^ | 0.41 ± 0.20 | 0.65 |  | 0.44 ± 0.21 | | 0.98 |  | -0.12 ± 0.21 | 0.92 |  | 0.03 ± 0.20 | 0.93 | |
|  | SWA | -0.16 ± 1.31 | 0.90 |  | 0.17 ± 1.33 | | 0.98 |  | -0.12 ± 0.21 | 0.95 |  | 1.17 ± 1.28 | 0.72 | |
|  | Intercept | -1.31 ± 1.18 | 0.70 |  | -1.47 ± 1.20 | | 0.98 |  | -1.13 ± 1.19 | 0.80 |  | 0.13 ± 1.16 | 0.93 | |
|  | SWA*BMI | 0.11 ± 0.12 | 0.70 |  | 0.13 ± 0.12 | | 0.98 |  | 0.06 ± 0.12 | 0.92 |  | 0.13 ± 00.11 | 0.68 | |
|  | SWA*Hypertension | 0.05 ± 0.43 | 0.70 |  | 0.33 ± 0.43 | | 0.98 |  | 0.03 ± 0.43 | 0.95 |  | 0.29 ± 0.42 | 0.88 | |
|  | SWA*Audit Class | 2.29 ± 3.25 | 0.70 |  | 1.54 ±3.31 | | 0.98 |  | 2.80 ± 3.27 | 0.85 |  | 3.98 ± 3.18 | 0.68 | |
|  | SWA*Diabetes | 0.48 ± 0.75 | 0.70 |  | 0.02 ± 0.77 | | 0.98 |  | -0.08 ± 0.76 | 0.95 |  | -1.15 ± 0.74 | 0.66 | |
|  | SWA*Education | 0.48 ± 0.39 | 0.70 |  | 0.33 ± 0.40 | | 0.98 |  | 0.08 ± 0.40 | 0.95 |  | 0.17 ± 0.38 | 0.93 | |
|  | SWA*Age | -0.07 ± 0.09 | 0.70 |  | -0.06 ± 0.09 | | 0.98 |  | 0.14 ± 0.09 | 0.64 |  | -0.10 ± 0.08 | 0.67 | |
|  | SWA*Sex | -0.25 ± 0.21 | 0.70 |  | -0.21 ± 0.21 | | 0.98 |  | -0.28 ± 0.21 | 0.64 |  | -0.34 ± 0.20 | 0.66 | |
|  | Model | F27,128=1.0; p =0.44;  R2=0.005, ∆ R^2^ = -0.045 | |  | F27,128=0.8; p =0.69;  R2=-0.03, ∆ R^2^ = 0.01 | | |  | F27,128=1.0; p =0.53;  R2=-0.007, ∆ R^2^ = -0.017 | |  | F27,128=1.3; p =0.16;  R2=0.05, ∆ R^2^ = 0.04 | | |
| Notes: Model: age, sex, education, BMI, hypertension, diabetes, and audit class, with interactions. ^#^ adjusted for multiple comparisons; ^ compared to men; high school certificate compared to university; ^+^ compared to those with diabetes; ^Y^heavy drinkers compared to abstainers. SDMT: Symbol Digit Modalities Test; DSBT: Digit-Span Backwards Task; PA: Physical activity; MVPA; moderate-to-vigorous physical activity. β are standardised. ∆ R^2^ compared to model presented in Table S1b | | | | | | | | | | | | | | |

Table S5a – The association between physical activity, measured by Physical Activity Recall survey, and memory and fluid intelligence when controlling for age, sex, education, BMI, hypertension, diabetes, and audit class with interaction terms.

|  |  | Memory | |  |  | Fluid Intelligence | |  |  | |
| --- | --- | --- | --- | --- | --- | --- | --- | --- | --- | --- |
|  |  | β ± SE | | p# |  | β ± SE | | p# |  | |
| Light PA (min∙week-1) | BMI | -0.11 ± 0.09 | | 0.92 |  | -0.07 ± 0.07 | | 0.99 |  | |
|  | Hypertension | 0.03 ± 0.52 | | 0.98 |  | 0.12 ± 0.40 | | 0.99 |  | |
|  | Audit class^Y^ | -0.32 ± 0.95 | | 0.92 |  | 0.10 ± 0.74 | | 0.99 |  | |
|  | Diabetes^+^ | -1.67 ± 3.73 | | 0.92 |  | -0.08 ± 2.93 | | 0.99 |  | |
|  | Education* | 0.68 ± 0.43 | | 0.92 |  | 0.60 ± 0.34 | | 0.99 |  | |
|  | Age | 0.05 ± 0.09 | | 0.92 |  | 0.04 ± 0.07 | | 0.99 |  | |
|  | Sex^ | 0.42 ± 0.18 | | 0.65 |  | 0.18 ± 0.14 | | 0.99 |  | |
|  | *PAR* | 5.05 ± 10.70 | | 0.92 |  | 0.11 ± 8.40 | | 0.99 |  | |
|  | Intercept | 0.73 ± 3.81 | | 0.95 |  | -0.69 ± 2.99 | | 0.99 |  | |
|  | *PAR* *BMI | 0.10 ± 0.12 | | 098 |  | 0.05 ± 0.09 | | 0.99 |  | |
|  | *PAR* *Hypertension | 0.40 ± 1.29 | | 0.92 |  | 0.66 ± 1.01 | | 0.99 |  | |
|  | *PAR* *Audit Class | 1.91 ± 1.26 | | 0.92 |  | 0.68 ± 0.99 | | 0.99 |  | |
|  | *PAR* *Diabetes | -7.00 ± 10.61 | | 0.92 |  | -0.99 ± 8.33 | | 0.99 |  | |
|  | *PAR* *Education | 0.36 ± 0.81 | | 0.98 |  | 0.18 ± 0.64 | | 0.99 |  | |
|  | *PAR* *Age | 0.13 ± 0.14 | | 0.92 |  | 0.07 ± 0.11 | | 0.99 |  | |
|  | *PAR* *Sex | -0.01 ± 0.28 | | 0.92 |  | -0.03 ± 0.22 | | 0.99 |  | |
|  | Model | F_27,128_=1.0 p =0.52;  R2=-0.006, ∆ R^2^ = -0.066 | | |  | F_27,128_=0.7 p =0.88;  R2=-0.06, ∆ R^2^ = -0.1 | | |  | |
| Moderate PA  ( min∙week-1) | BMI | -0.11 ± 0.09 | | 0.45 |  | -0.05 ± 0.07 | | 0.90 |  | |
|  | Hypertension | -0.13 ± 0.40 | | 0.83 |  | -0.09 ± 0.32 | | 0.90 |  | |
|  | Audit class^Y^ | 0.06 ± 0.88 | | 0.94 |  | 0.34 ± 0.70 | | 0.90 |  | |
|  | Diabetes^+^ | 2.37 ± 2.82 | | 0.54 |  | 0.44 ± 2.24 | | 0.90 |  | |
|  | Education* | 0.69 ± 0.40 | | 0.44 |  | 0.63 ± 0.32 | | 0.90 |  | |
|  | Age | 0.03 ± 0.08 | | 0.83 |  | 0.02 ± 0.07 | | 0.90 |  | |
|  | Sex^ | 0.42 ± 0.18 | | 0.44 |  | 0.02 ± 0.07 | | 0.90 |  | |
|  | *PAR* | -7.30 ± 6.96 | | 0.49 |  | -1.78 ± 5.53 | | 0.90 |  | |
|  | Intercept | -3.54 ± 2.92 | | 0.45 |  | -1.14 ± 2.32 | | 0.90 |  | |
|  | *PAR* *BMI | 0.05 ± 0.11 | | 0.83 |  | 0.04 ± 0.09 | | 0.90 |  | |
|  | *PAR* *Hypertension | -0.39 ± 9.44 | | 0.54 |  | -0.20 ± 0.35 | | 0.90 |  | |
|  | *PAR* *Audit Class | 3.48 ± 2.17 | | 0.44 |  | 1.53 ± 1.72 | | 0.90 |  | |
|  | *PAR* *Diabetes | 4.49 ± 6.76 | | 0.65 |  | 0.84 ± 5.37 | | 0.90 |  | |
|  | *PAR* *Education | 0.80 ± 0.65 | | 0.45 |  | 0.57 ± 0.51 | | 0.90 |  | |
|  | *PAR* *Age | -0.01 ± 0.08 | | 0.94 |  | 0.03 ± 0.07 | | 0.90 |  | |
|  | *PAR* *Sex | 0.36 ± 0.21 | | 0.44 |  | 0.25 ± 0.17 | | 0.90 |  | |
|  | Model | F_27,128_=1.2 p =0.25;  R2=0.03, ∆ R^2^ = -0.02 | | |  | F_27,128_=0.8 p =0.78;  R2=-0.04, ∆ R^2^ = -0.1 | | |  | |
| Vigorous PA  ( min∙week-1) | BMI | -0.11 ± 0.09 | | 0.72 |  | -0.04 ± 0.07 | | 0.98 |  | |
|  | Hypertension | -0.14 ± 0.52 | | 0.90 |  | -0.12 ± 0.41 | | 0.98 |  | |
|  | Audit class^Y^ | -11.00 ± 6.82 | | 0.61 |  | -9.32 ± 5.34 | | 0.73 |  | |
|  | Diabetes^+^ | 0.73 ± 0.71 | | 0.72 |  | 0.24 ± 0.55 | | 0.98 |  | |
|  | Education* | 0.71 ± 0.41 | | 0.61 |  | 0.68 ± 0.32 | | 0.73 |  | |
|  | Age | 0.02 ± 0.08 | | 0.90 |  | 0.02 ± 0.07 | | 0.98 |  | |
|  | Sex^ | 0.52 ± 0.19 | | 0.17 |  | 0.26 ± 0.15 | | 0.73 |  | |
|  | *PAR* | -0.42 ± 2.83 | | 0.91 |  | 0.08 ± 2.21 | | 0.98 |  | |
|  | Intercept | -0.86 ± 1.26 | | 0.72 |  | -0.67 ± 0.99 | | 0.98 |  | |
|  | *PAR* *BMI | 0.01 ± 0.16 | | 9,94 |  | 0.003 ± 0.13 | | 0.98 |  | |
|  | *PAR* *Hypertension | -0.24 ± 1.12 | | 0.90 |  | -0.22 ± 0.88 | | 0.98 |  | |
|  | *PAR* *Audit Class | 6.48 ± 5.69 | | 0.72 |  | 6.84 ± 4.45 | | 0.73 |  | |
|  | *PAR* *Diabetes | 1.44 ± 1.30 | | 0.72 |  | 0.43 ± 1.02 | | 0.98 |  | |
|  | *PAR* *Education | 0.48 ± 0.64 | | 0.72 |  | 0.17 ± 0.50 | | 0.98 |  | |
|  | *PAR* *Age | 0.11 ± 0.14 | | 0.72 |  | 0.13 ± 0.11 | | 0.84 |  | |
|  | *PAR* *Sex | 0.60 ± 0.32 | | 0.61 |  | 0.30 ± 0.25 | | 0.84 |  | |
|  | Model | F_27,128_=1.2 p =0.28;  R2=0.03, ∆ R^2^ = -0.02 | | |  | F_27,128_=0.9 p =0.62;  R2=-0.02, ∆ R^2^ = 0.06 | | |  | |
| MVPA  ( min∙week-1) | BMI | -0.12 ± 0.09 | | 0.50 |  | -0.04 ± 0.07 | | 0.95 |  | |
|  | Hypertension | -0.18 ± 0.41 | | 0.71 |  | -0.13 ± 1.64 | | 0.95 |  | |
|  | Audit class^Y^ | -1.45 ± 2.07 | | 0.59 |  | -1.37 ± 1.64 | | 0.95 |  | |
|  | Diabetes^+^ | 0.92 ± 0.74 | | 0.50 |  | 0.25 ± 0.59 | | 0.95 |  | |
|  | Education* | 0.69 ± 0.38 | | 0.41 |  | 0.63 ± 0.30 | | 0.76 |  | |
|  | Age | 0.02 ± 0.08 | | 0.87 |  | 0.01 ± 0.07 | | 0.95 |  | |
|  | Sex^ | 0.47 ± 0.18 | | 0.25 |  | 0.24 ± 0.14 | | 0.81 |  | |
|  | *PAR* | -4.40 ± 2.74 | | 0.50 |  | -1.05 ± 2.16 | | 0.95 |  | |
|  | Intercept | -2.35 ± 1.28 | | 0.41 |  | -0.91 ± 1.01 | | 0.95 |  | |
|  | *PAR* *BMI | 0.02 ± 0.11 | | 0.87 |  | 0.01 ± 0.09 | | 0.95 |  | |
|  | *PAR* *Hypertension | -0.28 ± 0.54 | | 0.68 |  | -0.13 ± 0.43 | | 0.95 |  | |
|  | *PAR* *Audit Class | 4.47 ± 2.93 | | 0.50 |  | 2.36 ± 2.32 | | 0.95 |  | |
|  | *PAR* *Diabetes | 1.62 ± 1.54 | | 0.50 |  | 0.54 ± 1.22 | | 0.95 |  | |
|  | *PAR* *Education | 0.60 ± 0.52 | | 0.50 |  | 0.26 ± 0.41 | | 0.95 |  | |
|  | *PAR* *Age | 0.07 ± 0.10 | | 0.59 |  | 0.10 ± 0.08 | | 0.95 |  | |
|  | *PAR* *Sex | 0.57 ± 0.24 | | 0.25 |  | 0.37 ± 0.19 | | 0.76 |  | |
|  | Model | F_27,128_=1.3 p =0.14  R2=0.06, ∆ R^2^ = 0.02 | | |  | F_27,128_=1.0 p =0.51  R2=-0.005, ∆ R^2^ = -0.045 | | |  | |
| TOTAL PA (MET:Min∙week-1) | BMI | -0.11 ± 0.09 | 0.37 | |  | -0.05 ± 0.07 | 0.98 | |  | |
|  | Hypertension | -0.25 ± 0.47 | 0.64 | |  | -0.16 ± 0.38 | 0.98 | |  | |
|  | Audit class^Y^ | -0.48 ± 1.59 | 0.79 | |  | -0.69 ± ± 1.26 | 0.98 | |  | |
|  | Diabetes^+^ | 0.96 ± 0.89 | 0.37 | |  | 0.25 ± 0.62 | 0.98 | |  | |
|  | Education* | 0.72 ± 0.39 | 0.35 | |  | 0.64 ± 0.31 | 0.98 | |  | |
|  | Age | 0.05 ± 0.08 | 0.64 | |  | 0.03 ± 0.07 | 0.98 | |  | |
|  | Sex^ | 0.47 ± 0.18 | 0.26 | |  | 0.23 ± 0.14 | 0.98 | |  | |
|  | *PAR* | -5.68 ± 3.25 | 0.35 | |  | -0.88 ± 2.58 | 0.98 | |  | |
|  | Intercept | -2.78 ± 1.37 | 0.35 | |  | -0.95 ± 1.09 | 0.98 | |  | |
|  | *PAR* *BMI | 0.01 ± 0.11 | 0.94 | |  | -0.001 ± 0.09 | 0.99 | |  | |
|  | *PAR* *Hypertension | -0.42 ± 0.78 | 0.64 | |  | -0.21 ± 0.62 | 0.98 | |  | |
|  | *PAR* *Audit Class | 5.12 ± 2.66 | 0.35 | |  | 1.60 ± 2.11 | 0.98 | |  | |
|  | *PAR* *Diabetes | 1.66 ± 1.82 | 0.35 | |  | 0.42 ± 1.44 | 0.98 | |  | |
|  | *PAR* *Education | 0.56 ± 0.57 | 0.35 | |  | 0.18 ± 9.45 | 0.98 | |  | |
|  | *PAR* *Age | 0.13 ± 0.12 | 0.41 | |  | 0.12 ± 0.09 | 0.98 | |  | |
|  | *PAR* *Sex | 0.46 ± 0.25 | 0.35 | |  | 0.26 ± 0.20 | 0.98 | |  | |
|  | Model | F_27,128_=1.3 p =0.19  R2=0.04, ∆ R^2^ = 0.00 | | |  | F_27,128_=0.8 p =0.70  R2=-0.03, ∆ R^2^ = 0.07 | | |  | |
| Notes: Model: age, sex, education, BMI, hypertension, diabetes, and audit class, with interactions. ^#^ adjusted for multiple comparisons; ^ compared to men; high school certificate compared to university; ^+^ compared to those with diabetes; ^Y^heavy drinkers compared to abstainers. SDMT: Symbol Digit Modalities Test; DSBT: Digit-Span Backwards Task; PA: Physical activity; MVPA; moderate-to-vigorous physical activity. β are standardised. ∆ R^2^ compared to model presented in Table S1a | | | | | | | | | |  |

Table S5b – The association between physical activity, measured by the SenseWear Armband™, and memory and fluid intelligence when controlling for age, sex, education, BMI, hypertension, diabetes, and audit class with interaction terms.

|  |  | Memory | |  |  | Fluid intelligence | | |  |  |
| --- | --- | --- | --- | --- | --- | --- | --- | --- | --- | --- |
|  |  | β ± SE | | p# |  | β ± SE | | | p# |  |
| Light PA (min∙week-1) | BMI | -0.11 ± 0.09 | | 0.79 |  | -0.06 ± 0.07 | | | 0.93 |  |
|  | Hypertension | -0.10 ± 0.38 | | 0.98 |  | -0.07 ± 0.30 | | | 0.93 |  |
|  | Audit class^Y^ | -0.62 ± 0.82 | | 0.79 |  | 0.06 ± 0.64 | | | 0.93 |  |
|  | Diabetes^+^ | 0.68 ± 0.69 | | 0.79 |  | 0.20 ± 0.54 | | | 0.93 |  |
|  | Education* | 0.60 ± 0.36 | | 0.76 |  | 0.58 ± 0.28 | | | 0.63 |  |
|  | Age | 0.02 ± 0.08 | | 0.98 |  | 0.01 ± 0.06 | | | 0.93 |  |
|  | Sex^ | 0.40 ± 0.17 | | 0.33 |  | 0.18 ± 0.14 | | | 0.93 |  |
|  | SWA | -0.002 ± 0.09 | | 0.98 |  | -0.01 ±0.07 | | | 0.93 |  |
|  | Intercept | -1.28 ± 1.00 | | 0.79 |  | -0.76 ± 0.78 | | | 0.93 |  |
|  | Model | F_14,141_=1.4; p =0.18;  R2=0.03, ∆ R^2^ = -0.02 | | |  | F_14,141_=1.0; p =0.50;  R2=-0.004, ∆ R^2^ = -0.044 | | | |  |
| Moderate PA ( min∙week-1) | BMI | -0.11 ± 0.09 | | 0.79 |  | -0.04 ± 0.07 | | | 0.98 |  |
|  | Hypertension | -0.10 ± 0.38 | | 0.98 |  | -0.7 ± 0.30 | | | 0.98 |  |
|  | Audit class^Y^ | -0.63 ± 0.84 | | 0.79 |  | -0.14 ± 0.65 | | | 0.98 |  |
|  | Diabetes^+^ | 0.67 ± 0.69 | | 0.79 |  | 0.19 ± 0.54 | | | 0.98 |  |
|  | Education* | 0.60 ± 0.36 | | 0.75 |  | 0.56 ± 0.28 | | | 0.98 |  |
|  | Age | 0.02 ± 0.08 | | 0.98 |  | 0.02 ± 0.14 | | | 0.98 |  |
|  | Sex^ | 0.40 ± 0.18 | | 0.43 |  | 0.20 ± 0.14 | | | 0.98 |  |
|  | SWA | 0.002 ± 0.09 | | 0.98 |  | 0.03 ± 0.07 | | | 0.98 |  |
|  | Intercept | -1.27 ± 0.99 | | 0.79 |  | -0.72 ± 0.77 | | | 0.98 |  |
|  | Model | F_14,141_=1.361; p =0.18;  R2=0.03, ∆ R^2^ = -0.01 | | |  | F_14,141_=0.9723; p =0.48;  R2=-0.003, ∆ R^2^ = -0.63 | | | |  |
| Vigorous PA ( min∙week-1) | BMI | -0.09 ± 0.09 | | 0.72 |  | -0.03 ± 0.07 | | | 0.94 |  |
|  | Hypertension | -0.08 ± 0.38 | | 0.92 |  | -0.05 ± 0.30 | | | 0.94 |  |
|  | Audit class^Y^ | -0.38 ± 0.51 | | 0.72 |  | 0.05 ± 0.63 | | | 0.94 |  |
|  | Diabetes^+^ | 0.68 ± 0.69 | | 0.72 |  | 0.20 ± 0.54 | | | 0.94 |  |
|  | Education* | 0.59 ± 0.36 | | 0.72 |  | 0.56 ± 0.28 | | | 0.71 |  |
|  | Age | 0.03 ± 0.08 | | 0.92 |  | 0.02 ± 0.06 | | | 0.94 |  |
|  | Sex^ | 0.43 ± 0.18 | | 0.24 |  | 0.21 ± 0.14 | | | 0.80 |  |
|  | SWA | 0.06 ± 0.09 | | 0.72 |  | 0.07 ± 0.07 | | | 0.80 |  |
|  | Intercept | -1.31 ± 0.99 | | 0.72 |  | -0.77 ± 0.77 | | | 0.80 |  |
|  | Model | F_14,141_=1.4; p =0.16;  R2=0.04, ∆ R^2^ = -0.01 | | |  | F_14,141_=1.0; p =0.41;  R2=0.004, ∆ R^2^ = -0.046 | | | |  |
| MVPA ( min∙week-1) | BMI | -0.10 ± 0.09 | | 0.77 |  | -0.04 ± 0.07 | | | 0.98 |  |
|  | Hypertension | -0.10 ± 0.38 | | 0.92 |  | -0.07 ± 0.30 | | | 0.98 |  |
|  | Audit class^Y^ | -0.65 ± 0.84 | | 0.77 |  | -0.04 ± 0.65 | | | 0.98 |  |
|  | Diabetes^+^ | 0.67 ± 0.69 | | 0.77 |  | 0.19 ± 0.54 | | | 0.98 |  |
|  | Education* | 0.59 ± 0.36 | | 0.77 |  | 0.56 ± 0.28 | | | 0.71 |  |
|  | Age | 0.02 ± 0.08 | | 0.92 |  | 0.02 ± 0.06 | | | 0.98 |  |
|  | Sex^ | 0.41 ± 0.18 | | 0.40 |  | 0.21 ± 0.14 | | | 0.98 |  |
|  | SWA | 0.02 ± 0.09 | | 0.92 |  | 0.05 ± 0.07 | | | 0.98 |  |
|  | Intercept | -1.27 ± 0.99 | | 0.77 |  | -0.72 ± 0.77 | | | 0.98 |  |
|  | Model | F_14,141_=1.4; p =0.18;  R2=0.03, ∆ R^2^ = -0.01 | | |  | F_14,141_=1.0; p =0.47;  R2=-0.001, ∆ R^2^ = -0.041 | | | |  |
| TOTAL PA (MET:mins∙week^-1^) | BMI | -0.09 ± 0.10 | 0.73 | |  | -0.02 ± 0.07 | 0.98 | | |  |
|  | Hypertension | -0.10 ± 0.38 | 0.94 | |  | -0.06 ± 0.30 | 0.98 | | |  |
|  | Audit class^Y^ | -0.69 ± 0.83 | 0.73 | |  | -0.06 ± 0.65 | 0.98 | | |  |
|  | Diabetes^+^ | 0.68 ± 0.69 | 0.73 | |  | 0.19 ± 9.54 | 0.98 | | |  |
|  | Education* | 0.58 ± 0.36 | 0.73 | |  | 0.34 ± 0.32 | 0.81 | | |  |
|  | Age | 0.03 ± 0.08 | 0.94 | |  | 0.02 ± 0.06 | 0.98 | | |  |
|  | Sex^ | 0.42 0.18 | 0.30 | |  | 0.22 ± 0.14 | 0.98 | | |  |
|  | SWA | 0.04 ± 0.10 | 0.94 | |  | 0.07 ± 0.07 | | 0.98 | |  |
|  | Intercept | -1.25 ± 0.99 | 0.73 | |  | -0.69 ± 0.77 | 0.98 | | |  |
|  | Model | F_14,141_=1.4; p =0.17;  R2=0.03, ∆ R^2^ = -0.02 | | |  | F_14,141_=1.0; p =0.44;  R2=0.002, ∆ R^2^ = -0.048 | | | |  |
| Notes: Model: age, sex, education, BMI, hypertension, diabetes, and audit class. ^#^ adjusted for multiple comparisons; ^ compared to men; high school certificate compared to university; ^+^ compared to those with diabetes; ^Y^heavy drinkers compared to abstainers. SDMT: Symbol Digit Modalities Test; DSBT: Digit-Span Backwards Task; PA: Physical activity; MVPA; moderate-to-vigorous physical activity. β are standardised. ∆ R^2^ compared to model presented in Table S1b | | | | | | | | | | |

Table S6 – The association between physical activity, measured by the SenseWear Armband™ relative to Q1, and cognitive function when controlling for age, sex, & education, with & without interaction terms.

|  |  | Immediate recall |  |  | Delayed recall |  |  | SDMT |  |  | DSBT |  |
| --- | --- | --- | --- | --- | --- | --- | --- | --- | --- | --- | --- | --- |
| MVPA ( min∙week-1) |  | β ± SE | p# |  | β ± SE | p# |  | β ± SE | p# |  | β ± SE | p# |
|  | Education^*^ | 0.81 ± 0.36 | 0.09 |  | 0.46 ± 0.37 | 0.63 |  | 0.45 ± 0.37 | 0.81 |  | 0.67 ± 0.37 | 0.45 |
|  | Age | 0.05 ± 0.08 | 0.52 |  | -0.03 ± 0.08 | 0.88 |  | -0.08 ± 0.08 | 0.81 |  | 0.09 ± 0.08 | 0.45 |
|  | Sex^ | 0.37 ± 0.17 | 0.09 |  | 0.37 ± 0.17 | 0.31 |  | -0.06 ± 0.17 | 0.81 |  | 0.04 ± 0.18 | 0.91 |
|  | SWA^2^ | 0.06 ± 0.23 | 0.81 |  | 0.09 ± 0.23 | 0.88 |  | -0.24 ± 0.23 | 0.81 |  | -0.26 ± 0.23 | 0.45 |
|  | SWA^3^ | -0.05 ± 0.23 | 0.81 |  | 0.07 ± 0.23 | 0.88 |  | 0.11 ± 0.23 | 0.81 |  | -0.13 ± 0.23 | 0.73 |
|  | SWA^4^ | 0.07 ± 0.24 | 0.81 |  | -0.07 ± 0.24 | 0.88 |  | 0.17 ± 0.24 | 0.81 |  | 0.01 ± 0.24 | 0.97 |
|  | Intercept | -0.89 ± 0.40 | 0.09 |  | -0.57 ± 0.40 | 0.63 |  | -0.29 ± 0.40 | 0.81 |  | -0.49 ± 0.41 | 0.45 |
|  | Model | F_8,147_=1.8; p =0.08;  R^2^=0.09 | |  | F_8,147_=1.7; p =0.11;  R^2^=-0.08 | |  | F_8,147_=1.2; p =0.30;  R^2^=-0.06 | |  | F_8,147_=0.9; p =0.51;  R^2^=-0.05 | |
| MVPA ( min∙week-1) | Education^*^ | 0.42 ± 0.61 | 0.78 |  | 0.15 ± 0.62 | 0.91 |  | 0.58 ± 0.62 | 0.83 |  | 1.11 ± 0.62 | 0.22 |
|  | Age | 0.30 ± 0.17 | 0.42 |  | 0.12 ± 0.17 | 0.91 |  | 0.58 ± 0.77 | 0.83 |  | 1.01 ± 0.77 | 0.39 |
|  | Sex^ | 0.72 ± 0.37 | 0.42 |  | 0.41 ± 0.38 | 0.91 |  | 0.06 ± 0.38 | 0.90 |  | 0.39 ± 0.38 | 0.39 |
|  | SWA^2^ | 0.61 ± 1.23 | 0.83 |  | -0.40 ± 1.25 | 0.91 |  | 0.40 ± 1.25 | 0.90 |  | 1.13 ± 1.12 | 0.42 |
|  | SWA^3^ | 0.57 0.91 | 0.78 |  | 0.52 ± 0.94 | 0.91 |  | 0.77 ± 0.93 | 0.83 |  | 1.89 ± 0.93 | 0.22 |
|  | SWA^4^ | -1.60 ± 1.19 | 0.44 |  | -1.91± 1.22 | 0.72 |  | 0.98 ± 1.21 | 0.83 |  | -1.91 ± 1.21 | 0.25 |
|  | Intercept | -1.00 ± 0.67 | 0.42 |  | -0.44 ± 0.69 | 0.91 |  | -0.55 ± 0.68 | 0.83 |  | -1.23 ± 0.68 | 0.22 |
|  | SWA^1^*Education^*^ | -0.19 ± 1.18 | 0.98 |  | 0.50 ± 1.20 | 0.91 |  | -0.61 ± 1.20 | 0.86 |  | -1.24 ± 1.20 | 0.39 |
|  | SWA^3^*Education^*^ | 0.08 ± 0.86 | 0.98 |  | -0.28 ± 0.87 | 0.91 |  | -0.61 ± 0.87 | 0.83 |  | -1.31 ± 0.87 | 0.25 |
|  | SWA^4^*Education^*^ | 2.38 ± 1.18 | 0.42 |  | 2.32 ± 1.21 | 0.72 |  | 1.74 ± 1.20 | 0.83 |  | 2.28 ± 1.20 | 0.22 |
|  | SWA^1^*Age | -0.35 ± 0.23 | 0.42 |  | -0.15 ± 0.24 | 0.91 |  | -0.05 ± 0.23 | 0.90 |  | -0.06 ± 0.23 | 0.80 |
|  | SWA^3^*Age | -0.57 ± 0.25 | 0.42 |  | -0.42 ± 0.26 | 0.72 |  | 0.41 ± 0.26 | 0.80 |  | -0.23 ± 0.26 | 0.42 |
|  | SWA^4^*Age | -0.27 ± 1.33 | 0.51 |  | -0.18 ± 0.24 | 0.91 |  | 0.39 ± 0.24 | 0.83 |  | -1.07 ± 1.23 | 0.22 |
|  | SWA^1^*Sex | -0.01 ± 0.50 | 0.98 |  | 0.32 ± 0.51 | 0.91 |  | -0.17 ± 0.50 | 0.90 |  | -1.24 ± 1.20 | 0.70 |
|  | SWA^3^* Sex | -0.63 ± 0.51 | 0.48 |  | -0.09 ± 0.52 | 0.91 |  | 0.02 ± 0.52 | 0.87 |  | -1.31 ± 0.87 | 0.25 |
|  | SWA^4^* Sex | -0.70 ± 0.53 | 0.44 |  | -0.54 ± 0.54 | 0.91 |  | -0.59 ± 0.53 | 0.83 |  | 2.28 ± 1.20 | 0.32 |
|  | Model | F_23,132_=1.5; p =0.09;  R^2^=0.2 | |  | F23,132=1.2; p =0.30;  R^2^=-0.17 | |  | F23,132=1.2; p =0.23;  R^2^=-0.18 | |  | F23, 132=1.3; p =0.21;  R^2^=0.18 | |
| Notes: Model: age, sex, education, BMI, hypertension, diabetes, and audit class, with interactions. ^#^ adjusted for multiple comparisons; ^ compared to men; high school certificate compared to university; ^+^ compared to those with diabetes; ^Y^heavy drinkers compared to abstainers. SDMT: Symbol Digit Modalities Test; DSBT: Digit-Span Backwards Task; PA: Physical activity; MVPA; moderate-to-vigorous physical activity. β are standardised. R^2^ is multiple | | | | | | | | | | | | |

Table S7 – The association between physical activity, measured by the SenseWear Armband™ relative to Q2, and cognitive function when controlling for age, sex, & education, with & without interaction terms.

|  |  | Immediate recall |  |  | Delayed recall |  |  | SDMT |  |  | DSBT |  |
| --- | --- | --- | --- | --- | --- | --- | --- | --- | --- | --- | --- | --- |
| MVPA ( min∙week-1) |  | β ± SE | p# |  | β ± SE | p# |  | β ± SE | p# |  | β ± SE | p# |
|  | Education^*^ | 0.81 ± 0.36 | 0.12 |  | 0.46 ± 0.37 | 0.71 |  | 0.45 ± 0.37 | 0.50 |  | 0.67 ± 0.37 | 0.33 |
|  | Age | 0.37 ± 0.17 | 0.78 |  | -0.03 ± 0.08 | 0.93 |  | 0.08 ± 0.08 | 0.52 |  | 0.09 ± 0.09 | 0.39 |
|  | Sex^ | 0.37 ± 0.17 | 0.12 |  | 0.37 ± 0.17 | 0.31 |  | -0.06 ± 0.17 | 0.81 |  | 0.04 ± 0.18 | 0.84 |
|  | SWA^1^ | -0.06 ± 0.23 | 0.90 |  | -0.09 ± 0.23 | 0.93 |  | 0.24 ± 0.23 | 0.52 |  | 0.26 ± 0.23 | 0.39 |
|  | SWA^3^ | -0.06 ± 0.23 | 0.80 |  | -0.02 ± 0.22 | 0.93 |  | 0.35 ± 0.23 | 0.50 |  | 0.12 ± 0.23 | 0.66 |
|  | SWA^4^ | 0.01 ± 0.22 | 0.95 |  | -0.16 ± 0.23 | 0.89 |  | 0.40 ± 0.23 | 0.50 |  | 0.26 ± 0.23 | 0.39 |
|  | Intercept | -0.83 ± 0.40 | 0.12 |  | -0.48 ± 0.40 | 0.71 |  | -0.53 ± 0.41 | 0.50 |  | -0.74 ± 0.41 | 0.33 |
|  | Model | F_8,147_=1.8; p =0.08;  R^2^=0.09 | |  | F_8,147_=1.7; p =0.11;  R^2^=-0.08 | |  | F_8,147_=1.2; p =0.30;  R^2^=-0.06 | |  | F_8,147_=0.9; p =0.51;  R^2^=-0.05 | |
| MVPA ( min∙week-1) | Education^*^ | -0.23 ± 1.01 | 0.98 |  | 0.65 ± 1.03 | 0.75 |  | -0.03 ± 1.03 | 0.99 |  | -0.12 1.02 | 0.95 |
|  | Age | -0.04 ± 0.16 | 0.98 |  | -0.04 ± 0.16 | 0.88 |  | -0.30 ± 0.16 | 0.53 |  | 0.12 ± 0.16 | 0.80 |
|  | Sex^ | 0.71 ± 0.33 | 0.38 |  | 0.73 ± 0.34 | 0.74 |  | -0.11 ± 0.33 | 0.96 |  | 0.18 ± 0.33 | 0.84 |
|  | SWA^1^ | -0.60 ± 1.23 | 0.98 |  | 0.40 ± 1.25 | 0.88 |  | -0.40 ± 1.25 | 0.96 |  | -1.13 ± 1.24 | 0.80 |
|  | SWA^3^ | -0.04 ± 1.20 | 0.98 |  | 0.92 ± 1.23 | 0.75 |  | 0.37 ± 1.22 | 0.96 |  | 0.76 ± 1.22 | 0.80 |
|  | SWA^4^ | -2.21 ± 1.42 | 0.64 |  | -1.51 ± 1.45 | 0.75 |  | 0.37 ± 1.22 | 0.96 |  | -3.04 ± 1.44 | 0.22 |
|  | Intercept | -0.39 ± 1.03 | 0.98 |  | -0.84 ± 1.05 | 0.75 |  | -0.15 ± 1.04 | 0.99 |  | -0.10 ± 1.04 | 0.95 |
|  | SWA^1^*Education^*^ | 0.19 ± 1.18 | 0.98 |  | -0.50 ± 1.20 | 0.88 |  | 0.61 ± 1.20 | 0.96 |  | 1.24 ± 0.20 | 0.80 |
|  | SWA^3^*Education^*^ | 0.27 ± 1.18 | 0.98 |  | -0.78 ± 1.20 | 0.75 |  | 0.003 ± 1.20 | 0.99 |  | -0.07 ± 1.19 | 0.95 |
|  | SWA^4^*Education^*^ | 2.56 ± 1.43 | 0.60 |  | 1.82 ± 1.46 | 0.75 |  | 2.35 ± 1.45 | 0.65 |  | 3.51 ± 1.45 | 0.20 |
|  | SWA^1^*Age | 0.35 ± 0.23 | 0.65 |  | 0.15 ± 0.24 | 0.75 |  | 0.05 ± 0.23 | 0.98 |  | 0.06 ± 0.23 | 0.95 |
|  | SWA^3^*Age | -0.22 ± 0.25 | 0.89 |  | -0.27 ± 0.25 | 0.75 |  | 0.47 ± 0.25 | 0.53 |  | -0.17 ± 0.25 | 0.80 |
|  | SWA^4^*Age | 0.08 ± 0.23 | 0.98 |  | -0.03 ± 0.23 | 0.89 |  | 0.44 ± 0.23 | 0.53 |  | 4.20 ± 1.56 | 0.98 |
|  | SWA^1^*Sex | 0.01 ± 0.50 | 0.98 |  | -0.32 ± 0.51 | 0.75 |  | 0.17 ± 0.50 | 0.96 |  | 0.21 ± 0.50 | 0.88 |
|  | SWA^3^* Sex | -0.62 ± 0.48 | 0.66 |  | -0.40 ± 0.49 | 0.75 |  | 0.19 ± 0.49 | 0.96 |  | -0.58 ± 0.49 | 0.80 |
|  | SWA^4^* Sex | -0.70 ± 0.49 | 0.66 |  | -0.85 ± 0.51 | 0.75 |  | -0.42 ± 0.50 | 0.96 |  | -0.48 ± 0.50 | 0.80 |
|  | Model | F_23,132_=1.5; p =0.09;  R^2^=0.02 | |  | F23,132=1.2; p =0.30;  R^2^=-0.17 | |  | F23,132=1.2; p =0.23;  R^2=^=0.18 | |  | F23, 132=1.3; p =0.21;  R^2^=0.18 | |
| Notes: Model: age, sex, education, BMI, hypertension, diabetes, and audit class, with interactions. ^#^ adjusted for multiple comparisons; ^ compared to men; high school certificate compared to university; ^+^ compared to those with diabetes; ^Y^heavy drinkers compared to abstainers. SDMT: Symbol Digit Modalities Test; DSBT: Digit-Span Backwards Task; PA: Physical activity; MVPA; moderate-to-vigorous physical activity. β are standardised. R^2^ is multiple | | | | | | | | | | | | |

Table S8 – The association between physical activity, measured by the SenseWear Armband™ relative to Q3, and cognitive function when controlling for age, sex, & education, with & without interaction terms.

|  |  | Immediate recall |  |  | Delayed recall |  |  | SDMT |  |  | DSBT |  |
| --- | --- | --- | --- | --- | --- | --- | --- | --- | --- | --- | --- | --- |
| MVPA ( min∙week-1) relative to 3^rd^ quartile |  | β ± SE | p# |  | β ± SE | p# |  | β ± SE | p# |  | β ± SE | p# |
|  | Education^*^ | 0.81 ± 0.36 | 0.09 |  | 0.46 ± 0.37 | 0.63 |  | 0.45 ± 0.37 | 0.82 |  | 0.67 ± 0.37 | 0.52 |
|  | Age | 0.05 ± 0.08 | 0.70 |  | -0.03 ± 0.08 | 0.93 |  | -0.08 ± 0.08 | 0.82 |  | 0.09 ± 0.08 | 0.54 |
|  | Sex^ | 0.37 ± 0.17 | 0.09 |  | 0.37 ± 0.17 | 0.31 |  | -0.06 ± 0.17 | 0.82 |  | 0.04 ± 0.18 | 0.80 |
|  | SWA^1^ | 0.05 ± 0.23 | 0.81 |  | -0.07 ± 0.23 | 0.93 |  | -0.11 ± 0.23 | 0.82 |  | 0.13 ± 0.23 | 0.66 |
|  | SWA^2^ | 0.11 ± 0.22 | 0.70 |  | 0.02 ± 0.22 | 0.93 |  | -0.35 ± 0.23 | 0.82 |  | -0.12 ± 0.23 | 0.66 |
|  | SWA^4^ | 0.12 ± 0.23 | 0.70 |  | -0.14 ± 0.23 | 0.93 |  | 0.05 ± 0.23 | 0.82 |  | 0.14 ± 0.23 | 0.66 |
|  | Intercept | -0.95 ± 0.39 | 0.90 |  | -0.50 ± 0.39 | 0.63 |  | -0.18 ± 0.39 | 0.82 |  | -0.63 ± 0.40 | 0.52 |
|  | Model | F_8, 147_=1.8; p =0.08;  R^2^=0.09 | |  | F_8, 147_=1.8; p =0.08;  R^2^=0.09 | |  | F_8, 147_=1.2; p =0.20;  R^2^=0.06 | |  | F_8, 147_=0.9; p =0.51;  R^2^=0.05 | |
| MVPA ( min∙week-1) relative to 3^rd^ quartile interactions | Education^*^ | 0.50 ± 0.60 | 0.89 |  | -0.13 ± 0.62 | 0.90 |  | -0.03 ± .61 | 0.99 |  | -0.20 ± 0.61 | 0.85 |
|  | Age | -0.26 ± 0.19 | 0.61 |  | -0.30 ± 0.20 | 0.49 |  | 0.16 ± 0.19 | 0.88 |  | -0.04 ± 0.19 | 0.88 |
|  | Sex^ | 0.09 ± 0.35 | 0.99 |  | 0.33 ± 0.36 | 0.78 |  | 0.08 ± 0.35 | 0.99 |  | -0.40 ± 0.35 | 0.48 |
|  | SWA^1^ | -0.57 ± 0.91 | 0.94 |  | -0.52 ± 0.94 | 0.82 |  | -0.77 ± 0.93 | 0.88 |  | -1.89 ± 0.93 | 0.15 |
|  | SWA^2^ | 0.04 ± 1.20 | 0.99 |  | -0.92 ± 1.23 | 0.78 |  | -0.37 ± 1.22 | 0.99 |  | -0.76 ± 1.21 | 0.64 |
|  | SWA^4^ | -2.16 ± 1.16 | 0.39 |  | -2.43 ± 1.19 | 0.42 |  | -1.74 ± 1.18 | 0.68 |  | -3.78 ± 1.18 | 0.12 |
|  | Intercept | -0.43 ± 0.62 | 0.94 |  | 0.08 ± 0.64 | 0.90 |  | 0.21 ± 0.63 | 0.99 |  | 0.65 ± 0.63 | 0.52 |
|  | SWA^1^*Education^*^ | -0.08 ± 0.86 | 0.99 |  | 0.28 ± 0.87 | 0.90 |  | -0.02 ± 0.52 | 0.88 |  | 1.31 ± 0.87 | 0.29 |
|  | SWA^3^*Education^*^ | -0.27 ± 1.18 | 0.99 |  | 0.78 ± 0.12 | 0.78 |  | -0.77 ± 0.69 | 0.99 |  | 0.07 ± 1.19 | 0.95 |
|  | SWA^4^*Education^*^ | 2.29 ± 1.18 | 0.39 |  | 2.60 ± 1.20 | 0.42 |  | -0.03 ± 0.61 | 0.68 |  | 3.59 ± 1.20 | 0.02 |
|  | SWA^1^*Age | 0.57 ± 0.25 | 0.39 |  | 0.42 ± 0.26 | 0.49 |  | -0.41 ± 0.26 | 0.68 |  | 0.23 ± 0.26 | 0.60 |
|  | SWA^3^*Age | 0.22 ± 0.25 | 0.89 |  | 0.27 ± 0.25 | 0.78 |  | -0.47 ± 0.25- | 0.99 |  | 0.17 ± 0.25 | 0.64 |
|  | SWA^4^*Age | 0.30 ± 0.25 | 0.61 |  | 0.24 ± 0.26 | 0.78 |  | -0.02 ± 0.26 | 0.99 |  | -0.19 ± 0.25 | 0.64 |
|  | SWA^1^*Sex | 0.63 ± 0.51 | 0.61 |  | 0.09 ± 0.52 | 0.90 |  | -0.02 ± 0.52 | 0.99 |  | 0.29 ± 0.52 | 0.29 |
|  | SWA^3^* Sex | 0.62 ± 0.48 | 0.61 |  | 0.40 ± 0.49 | 0.78 |  | -0.19 ± 0.49 | 0.81 |  | 0.58 ± 0.49 | 0.47 |
|  | SWA^4^* Sex | -0.07 ± 0.51 | 0.99 |  | -0.45 ± 0.52 | 0.78 |  | -0.61 ± 0.52 | 0.68 |  | 0.10 ± 0.52 | 0.88 |
|  | Model | F_23,132_=1.5; p =0.09;  R^2^=0.2 | |  | F_23,132_=1.2; p =0.30;  R^2^=0.2 | |  | F23,132=1.2; p =0.23;  R^2^=-0.18 | |  | F23, 132=1.3; p =0.21;  R^2^=0.18 | |
| Notes: Model: age, sex, education, BMI, hypertension, diabetes, and audit class, with interactions. ^#^ adjusted for multiple comparisons; ^ compared to men; high school certificate compared to university; ^+^ compared to those with diabetes; ^Y^heavy drinkers compared to abstainers. SDMT: Symbol Digit Modalities Test; DSBT: Digit-Span Backwards Task; PA: Physical activity; MVPA; moderate-to-vigorous physical activity. β are standardised. R^2^ is multiple | | | | | | | | | | | | |

Table S9 – The association between physical activity, measured by the SenseWear Armband™ relative to Q3, and cognitive function when controlling for age, sex, & education, with & without interaction terms.

|  |  | Immediate recall |  |  | Delayed recall |  |  | SDMT |  |  | DSBT |  |
| --- | --- | --- | --- | --- | --- | --- | --- | --- | --- | --- | --- | --- |
| MVPA ( min∙week-1) relative to 4^th^ quartile |  | β ± SE | p# |  | β ± SE | p# |  | β ± SE | p# |  | β ± SE | p# |
|  | Education^*^ | 0.81 ± 0.36 | 0.11 |  | 0.46 ± 0.37 | 0.63 |  | 0,45 0.37 | 0.82 |  | 0.67 ± 0.37 | 0.45 |
|  | Age | 0.05 ± 0.08 | 0.75 |  | -0.03 ± 0.08 | 0.88 |  | -0.08 ± 0.08 | 0.82 |  | 0.09 ± 0.08 | 0.45 |
|  | Sex^ | 0.37 ± 0.17 | 0.11 |  | 0.27 ± 0.17 | 0.31 |  | -0.06 ± 0.17 | 0.82 |  | 0.04 ± 0.18 | 0.91 |
|  | SWA^1^ | -0.07 ± 0.24 | 0.86 |  | 0.07 ± 0.24 | 0.88 |  | -0.17 ± 0.24 | 0.82 |  | -0.01 ± 0.24 | 0.92 |
|  | SWA^2^ | -0.01 ± 0.23 | 0.95 |  | 0.16 ± 0.23 | 0.83 |  | -0.40 ± 0.23 | 0.76 |  | -0.26 ± 0.23 | 0.45 |
|  | SWA^3^ | -0.12 ± 0.23 | 0.75 |  | 0.14 ± 0.23 | 0.83 |  | -0.05 ± 0.23 | 0.82 |  | -0.14 ± 0.23 | 0.71 |
|  | Intercept | -0.82 ± 0.39 | 0.11 |  | -0.64 ± 0.39 | 0.48 |  | -0.12 ± 0.40 | 0.82 |  | -0.48 ± 0.40 | 0.45 |
|  | Model | F_8, 147_=1.8; p =0.08;  R^2^=0.09 | |  | F_8, 147_=1.7; p =0.11;  R^2^=0.08 | |  | F_8, 147_=1.2; p =0.30;  R^2^=0.06 | |  | F_8_, _147_=0.9; p =0.51;  R^2^=0.05 | |
| MVPA ( min∙week-1) relative to 4^th^ quartile interactions | Education^*^ | 2.17 ± 1.01 | 0.11 |  | 2.47 ± 1.03 | 0.21 |  | 2.31 ± 1.03 | 0.39 |  | 3.39 ± 1.03 | 0.01 |
|  | Age | 0.04 ± 0.16 | 0.89 |  | -0.07 ± 0.17 | 0.75 |  | 0.14 ± 0.16 | 0.51 |  | -0.23 ± 0.16 | 0.20 |
|  | Sex^ | 0.02 ± 0.37 | 0.96 |  | -0.12 ± 0.38 | 0.78 |  | -0.53 ± 0.38 | 0.39 |  | -0.30 ± 0.38 | 0.46 |
|  | SWA^1^ | 1.60 ± 1.19 | 0.26 |  | 1.92 1.22 | 0.24 |  | 0.98 ± 1.21 | 0.51 |  | 1.91 ± 1.21 | 0.16 |
|  | SWA^2^ | 2.21 ± 1.42 | 0.34 |  | 1.51 ± 1.45 | 0.45 |  | 1.38 ± 1.44 | 0.51 |  | 3.04 ± 1.44 | 0.07 |
|  | SWA^3^ | 2.16 ± 1.16 | 0.17 |  | 2.43 ± 1.19 | 0.21 |  | 1.74 ± 1.18 | 0.39 |  | 3.80 ± 1.18 | 0.01 |
|  | Intercept | -2.59 ± 0.98 | 0.11 |  | -2.34 ± 1.00 | 0.21 |  | -1.52 ± 1.00 | 0.39 |  | -3.14 ± 1.00 | 0.01 |
|  | SWA^1^*Education^*^ | -2.38 ± 1.18 | 0.17 |  | -2.32 ± 1.28 | 0.21 |  | -1.74 ± 1.20 | 0.39 |  | -2.28 ± 1.20 | 0.10 |
|  | SWA^3^*Education^*^ | -2.56 ± 1.42 | 0.18 |  | -1.82 ± 1,46 | 0.34 |  | -2.35 ± 1.45 | 0.39 |  | -3.51 ± 1.45 | 0.04 |
|  | SWA^4^*Education^*^ | -2.29 ± 1.18 | 0.17 |  | -2.60 ± 1.20 | 0.21 |  | -2.34 ± 2.20 | 0.39 |  | -3.59 ± 1.20 | 0.01 |
|  | SWA^1^*Age | 0.27 ± 0.23 | 0.31 |  | 0.18 ± 0.24 | 0.54 |  | -0.39 ± 0.24 | 0.39 |  | 0.42 ± 0.24 | 0.85 |
|  | SWA^3^*Age | -0.08 ± 0.23 | 0.82 |  | 0.03 ± 0.23 | 0.90 |  | 0.42 ± 0.50 | 0.39 |  | 0.36 ± 0.23 | 0.12 |
|  | SWA^4^*Age | -0.30 ± 0.25 | 0.29 |  | -0.24 ± 0.26 | 0.47 |  | 0.61 ± 0.52 | 0.93 |  | 0.19 ± 0.25 | 0.16 |
|  | SWA^1^*Sex | 0.70 ± 0.53 | 0.26 |  | 0.54 ± 0.54 | 0.45 |  | 0.59 ± 0.53 | 0.43 |  | 0.69 ± 0.53 | 0.24 |
|  | SWA^3^* Sex | 0.69 ± 0.49 | 0.26 |  | 0.85 ± 0.51 | 0.22 |  | 0.42 ± 0.50 | 0.51 |  | 0.48 ± 0.50 | 0.84 |
|  | SWA^4^* Sex | 0.07 ± 0.51 | 0.93 |  | 0.45 ± 0.52 | 0.49 |  | 0.61 ± 0.52 | 0.42 |  | -0.10 ± 0.52 | 0.39 |
|  | Model | F_23,132_=1.5; p =0.09;  R^2^=0.2 | |  | F_23,132_=1.2; p =0.30;  R^2^=0.2 | |  | F_23,132_=1.2; p =0.23;  R^2^=-0.18 | |  | F_23, 132=_1.3; p =0.21;  R^2^=0.18 | |
| Notes: Model: age, sex, education, BMI, hypertension, diabetes, and audit class, with interactions. ^#^ adjusted for multiple comparisons; ^ compared to men; high school certificate compared to university; ^+^ compared to those with diabetes; ^Y^heavy drinkers compared to abstainers. SDMT: Symbol Digit Modalities Test; DSBT: Digit-Span Backwards Task; PA: Physical activity; MVPA; moderate-to-vigorous physical activity. β are standardised. R^2^ is multiple | | | | | | | | | | | | |
